# Computational Characterization of the mtORF of Pocilloporid Corals: Insights into Protein Structure and Function in *Stylophora* Lineages from Contrasting Environments

**DOI:** 10.3390/genes10050324

**Published:** 2019-04-27

**Authors:** Eulalia Banguera-Hinestroza, Evandro Ferrada, Yvonne Sawall, Jean-François Flot

**Affiliations:** 1 Evolutionary Biology and Ecology, Université libre de Bruxelles, B-1050 Brussels, Belgium; 2 Interuniversity Institute of Bioinformatics in Brussels—(IB)2, 1050 Brussels, Belgium; 3 Center for Genomics and Bioinformatics, Universidad Mayor, Santiago, Chile; evandro.ferrada@mayor.cl; 4 Coral Reef Ecology, Bermuda Institute of Ocean Sciences (BIOS), St.George’s GE 01, Bermuda; yvonne.sawall@bios.edu

**Keywords:** mtORF, *Stylophora*, adaptation, disordered residues, tandem repeats, pocilloporid corals, selection, transmembrane proteins

## Abstract

More than a decade ago, a new mitochondrial Open Reading Frame (mtORF) was discovered in corals of the family Pocilloporidae and has been used since then as an effective barcode for these corals. Recently, mtORF sequencing revealed the existence of two differentiated *Stylophora* lineages occurring in sympatry along the environmental gradient of the Red Sea (18.5 °C to 33.9 °C). In the endemic Red Sea lineage *RS_LinB*, the mtORF and the heat shock protein gene *hsp70* uncovered similar phylogeographic patterns strongly correlated with environmental variations. This suggests that the mtORF too might be involved in thermal adaptation. Here, we used computational analyses to explore the features and putative function of this mtORF. In particular, we tested the likelihood that this gene encodes a functional protein and whether it may play a role in adaptation. Analyses of full mitogenomes showed that the mtORF originated in the common ancestor of *Madracis* and other pocilloporids, and that it encodes a transmembrane protein differing in length and domain architecture among genera. Homology-based annotation and the relative conservation of metal-binding sites revealed traces of an ancient hydrolase catalytic activity. Furthermore, signals of pervasive purifying selection, lack of stop codons in 1830 sequences analyzed, and a codon-usage bias similar to that of other mitochondrial genes indicate that the protein is functional, i.e., not a pseudogene. Other features, such as intrinsically disordered regions, tandem repeats, and signals of positive selection particularly in *Stylophora* *RS_LinB* populations, are consistent with a role of the mtORF in adaptive responses to environmental changes.

## 1. Introduction

The mitochondrial genome of metazoans (multicellular animals) usually contain 37 genes with no introns, among which only 13 are protein coding [1]. However, deviations from this rule exists and new functional mitochondrial open reading frames (mtORFs) have been reported in several marine taxa, such as brachiopods [2], molluscs [3,4,5], cnidarians [6], and sponges (reviewed in [7]). In some cases, these new mtORFs have been shown to encode proteins with a genetic variability sufficient to unravel patterns of differentiation in marine populations and species from different geographic origins (e.g., in unicellular algae of the class Raphidophyceae [8]). 

In corals, a novel mtORF was reported by Flot and Tillier [9] in three genera of the family Pocilloporidae (*Pocillopora*, *Seriatopora* and *Stylophora*). This mtORF gene has been useful for the delimitation of species within *Pocillopora* [10,11] and has allowed the identification of cryptic species and of fine-scale genetic structure in *Seriatopora* [12,13,14]. Furthermore, it has revealed strong phylogenetic and phylogeographic patterns in *Stylophora* [15,16]. This indicates that in contrast to the absence of highly variable mtDNA genes in most corals [17,18], the mtORF may be a suitable mitochondrial barcode gene for pocilloporids [9,11], revealing genetic variation useful for distinguishing lineages evolving under different environmental conditions.

mtDNA genes, in particular barcode genes (e.g., the cytochrome *c* oxidase subunit I gene *cox1* [19]), often unravel patterns of genetic variation that are highly correlated with changes in environmental conditions, mostly related to temperature and altitude [19,20,21,22,23]. This is because they are involved in key molecular mechanisms related to mitochondrial bioenergetics. For example, they are key players in the mito-nuclear protein complexes of the oxidative phosphorylation (OXPHOS) system, which transforms ADP (adenosine 5′-diphosphate) and phosphate into the cellular energy carrier adenosine 5′-triphosphate (ATP) [24,25]. Hence, mtDNA genes play an important role in the thermal tolerance of organisms by regulating the availability of ATP, the demand of which is often increased under environmental extremes [26,27,28,29]. 

Positively selected mutations in mtDNA OXPHOS have been found to have an important impact on the ecology and evolutionary history of multiple taxa [20,21,22,27,30,31,32]. These mutations produce mito-nuclear incompatibilities between genotypes adapted to markedly different environments, creating reproductive barriers and increasing genetic differentiation among populations (particularly in species distributed across broad geographical ranges), which are some of the main processes known to lead to speciation [19,27,33,34,35]. 

The involvement of mtDNA genes in adaptation to the environment and in speciation seems to be supported by the evolutionary patterns revealed by the mtORF barcode gene in the genus *Stylophora*. In 2018, Banguera-Hinestroza et al. [15] analyzed 827 samples of *Stylophora* covering a broad geographical range of this genus, including the full latitudinal (12 degrees of latitude) and environmental gradients of the Red Sea. They found a high number of mutational changes in the mtORF as well as in its adjacent genes *nd6* and *atp6*. Interestingly, these genes are part of an apparently recombinant region that strongly differentiates a putative hybrid lineage (*RS_LinA*) from its sympatric parental species (*RS_LinB*), both inhabiting the entire environmental gradient of the Red Sea. Furthermore, in *RS_LinB*, the mtORF uncovered the existence of two well-differentiated populations restricted respectively to the colder northern regions or to the warmer central-southern Red Sea. The same phylogeographic pattern was observed for the *hsp70* gene [15], which encodes a heat-shock protein well known for its role in stress response and climatic adaptation [36,37,38,39,40]. Results from Banguera-Hinestroza et al. [15] therefore suggest that the mtORF as well as *hsp70* may have both been involved in the adaptation of the ancestral and endemic *Stylophora* lineage to the different environmental regimes of the Red Sea, including extremely warm conditions in the Southern region.

On a broad geographical scale, pocilloporid corals are found in tropical areas worldwide. Confirmed records indicate that *Stylophora* and *Seriatopora* are restricted to the Pacific and Indian oceans, while *Madracis* and *Pocillopora* have wider distributions and are also found in the Atlantic Ocean and the Caribbean Sea [41]. *Stylophora* lineages occur throughout the entire Red Sea in regions with strong environmental differences. The northernmost region of the Red Sea (Gulf of Aqaba, 32°N) is characterized by the coldest temperature regime (18.5 °C–28.8 °C), highest salinity (41 PSU) and generally lowest nutrient input, while the southernmost region (Farasan islands, 20°N) is characterized by the warmest temperature regime (23.5 °C–33.9 °C), lowest salinity (37 PSU) and highest nutrient input [42,43,44]. Furthermore, the multiple environmental and geological changes recorded in the Red Sea since its formation around 30–14 Mya [45,46] have strongly influenced the patterns of endemism and diversification of its fauna [47,48]. Some of these changes include hypersaline conditions, extreme variations in sea levels, extended periods of isolation from the Indian Ocean, and strong temperature fluctuations (up to 35 °C) [49,50]. The latter is considered an extreme condition for the growth and survival of coral reefs [43,51]. 

As mentioned above, there is mounting evidence that the mtORF reveals consistent phylogenetic and phylogeographic patterns in pocilloporids as well as patterns of population structure linked to environmental conditions, as barcode genes do. However, this mitochondrial region has been suggested to be a pseudogene [52,53], a hypothesis that has not been tested so far. Testing this hypothesis might help us elucidate whether evolutionary changes in the mtORF recapitulate adaptation and speciation in pocilloporids. Therefore, the aim of the current study was twofold: (*a*) to test whether the mtORF encodes and expresses a functional protein, by investigating the mtORF origin and exploring clues about its putative structure and function in pocilloporids; and (*b*) to search for signatures of selection in the mtORF of *Stylophora* species inhabiting a broad range of environmental conditions in the Red Sea. 

If not functional or expressed, long ORFs (more than 600 bp in the present case) are expected to accumulate mutations [54]. Indeed, one of the clearest signals of pseudogenization in a putative ORF is the presence of multiple stop codons or frameshift mutations [55,56]. Hence, as a first step we analyzed the hundreds of available mtORF sequences from *Pocillopora*, *Seriatopora,* and *Stylophora*, in search for stop codons. In addition, we performed analyses of codon usage bias and tested for signatures of natural selection in representative pocilloporid sequences. 

As a second step, we carried out a whole mitogenome comparative analysis to identify the first appearance of this mtORF in the evolutionary history of scleractinian corals, and used computational methods [57,58,59,60] to analyze its structure and function in *Madracis*, *Pocillopora, Seriatopora*, and *Stylophora*, including the two *Stylophora* lineages previously identified in the Red Sea [15]. Computational methods have been shown to predict reliably protein structure and function in non-model organisms [61]. Finally, we tested whether the mtORF may be involved in local adaptation by searching for signals of positive selection in the sequences of *Stylophora* lineages, particularly those distributed over a wide temperature gradient in the Red Sea.

## 2. Materials and Methods 

### 2.1. Evolutionary Origin of the mtORF

Complete mitochondrial genomes of all pocilloporids (*sensu* [62]), including *Madracis mirabilis* (NC011160), *Stylophora pistillata* (accession: EU400214), *Seriatopora hystrix* (accession: EF633600), *Seriatopora caliendrum* (EF633601), *Pocillopora damicornis* (accession: EU400213), and *Pocillopora eydouxi* (accession: EF526303) were downloaded from the NCBI database and aligned with mitogenomes of the two Red Sea *Stylophora* lineages identified in [15] (Supplementary Data S1): the endemic *Stylophora* lineage from the Red Sea (*RS_LinB*) and its putative hybrid with *S. pistillata* (*RS_linA*).

Full mitogenome alignments were performed using the software LASTZ [63] and visualized using AliTV [64]. The alignments were contrasted against a phylogeny of pocilloporids built using the most variable mitochondrial genes in these species (*nd2*, *nd6*, *atp6*, mtORF, and *nd4*) [15]. This phylogenetic tree was constructed using the maximum-likelihood approach implemented in PhyML [65] available at http://www.phylogeny.fr/index.cgi [66,67]. The genera *Madrepora*, *Polycyathus* and *Astrangia* were included as outgroups, following the scleractinian phylogeny of Chuang et al. [68]. Branch support was evaluated using the approximate Likelihood Ratio Test (aLRT) [69].

### 2.2. Characterization of the mtORF Gene

To test for signals of pseudogenization, a total of 1830 mtORF nucleotide sequences (of complete and partial lengths) of *Pocillopora*, *Seriatopora,* and *Stylophora* from the NCBI database, including more than 600 unpublished sequences, were translated using the coelenterate mitochondrial code in the program MEGA [70] and scanned for stop codons (accession numbers are provided in Supplementary Table S1). The start and end codons of the putative mtORF were identified by comparison with the protein sequences reported by Flot and Tillier [9]. To ensure the reliability of the data, sequences of *Stylophora* and *Pocillopora* from the Red Sea were taken exclusively from our collection [15,71]. Furthermore, we used the program Tandem Repeats Finder [72] to search for repeats in representative sequences. 

### 2.3. Annotation of Protein Domains and Detection of Intrinsic Disorder

mtORF sequences extracted from full mitochondrial genomes (see above; Supplementary Data S2) were analyzed using a set of protein prediction approaches available at the PSIPRED Protein Sequence Analysis Workbench (www.bioinf.cs.ucl.ac.uk/psipred [73]) and the InterProScan data base [59,74,75]. These methods allow testing for transmembrane domains (TMDs) and different degrees of disorder in protein sequences. 

The presence of TMDs was investigated using several predictors, including: (*i*) TMHMM Server 2.0, which predicts transmembrane helices based on a Hidden Markov Model (HMM). This method returns the posterior probabilities that each residue sits inside of the cell, outside of it or in a transmembrane helix (TMH), with low probabilities meaning weak support for TMHs [57,58]; (*ii*) the PSIPRED approach (Protein Structure Prediction) by Jones [76], a highly accurate method that uses two feed-forward neural networks to predict secondary structure in outputs generated using PSI-BLAST (Position-Specific Iterative Basic Local Alignment Search Tool) [60,77]; (*iii*) the MEMSAT3 method (MEMbrane protein Structure And Topology 3), which predicts the secondary structure of transmembrane protein using multiple alignments produced by PSI-BLAST and by scoring log-likelihoods ratios through different topological models to gather the consensus [61,73,78,79]; and (*iv*) the MEMSAT-SVM approach using Support Vectors Machines, which are binary classifiers used to categorize residue preferences before combining them into a probabilistic framework [61]. 

To distinguish TM regions from signal peptides (SP), we used signaIP 4.1 [80,81], available at www.expasy.org. Furthermore, the likelihood of a transmembrane helix being involved in the formation of pore-lining regions was calculated using MEMSAT. These regions run parallel to transmembrane helices and are vital for biological processes such as the transport of ions and molecules across membranes [61].

Disordered regions were identified using the consensus of several methods including DISOPRED [82,83,84], IUpred2A [85], and MobiDB [86]. DISOPRED implements a neural network combined with support vector machines (SVM), and in addition to disordered regions, it also provides information about possible binding of intrinsically disordered proteins to substrates. IUPred is a knowledge-based approach that provides a per-residue profile of the degree of disorder. In contrast, MobiDB integrates annotations of disordered regions through several other databases and methods [86]. For all methods, probabilities larger than 0.5 correspond to 95% confidence of identifying a true positive disordered region.

### 2.4. Structural and Functional Annotation

In order to identify homologs of mtORF among sequences of known structure and/or function, we used HMMER (http://www.ebi.ac.uk/Tools/hmmer) [87] and the FFAS server (http://ffas.godziklab.org/) [88]. HMMER uses profile HMMs to explore multiple profile data bases such as Pfam [89]. This approach has a high sensitivity for detecting remote homologs. Furthermore, a multiple-sequence alignment was build using Promals [90] and input to the FFAS server. The FFAS method integrates PSI-BLAST [91] and fold-recognition methods to search homologous proteins in several databases of model organisms, including Pfam [89], SCOP [92] and the non-redundant database (nr85), which contains sequence information from several sources [93,94].

Functional annotation was performed using FFPred 2.0 [95], which relies on the GOA (Gene Ontology Annotation data base) to predict Gene Ontology (GO) terms. The GO classification distinguishes among macromolecular interactions, biological processes, molecular function and cellular components. The FFPred method reports the posterior probability of a functional annotation in terms of reliability, which includes sensitivity, specificity and precision [96], by searching throughout a large data set of already known proteins and annotations from eukaryotes [95]. 

Furthermore, the prediction of structural domains for mtORF sequence of each genus and lineage was carried out using pDomTHREADER [97]. This method retrieves structural hits with CATH domains annotated in the CATH protein data base using sequence and structural data [98]. The confidence in the existence of a given domain in the query proteins is reflected by its *p*-values. Low hits correspond to *p*-values ≤ 0.1, medium hits to *p*-values ≤ 0.01, high hits to *p*-values ≤ 0.001 and highly accurate hits to *p*-values ≤ 0.0001 [60].

### 2.5. Signatures of Selection in a Family Framework and Analysis of Codon Usage 

#### 2.5.1. Selection

The hypothesis that the mtORF of pocilloporid corals encodes a functional protein was also tested by performing an evolutionary rate analysis across species. If this hypothesis is true, some form of selection should be observed in the mtORF-encoded putative protein. For instance, the effect of the constraints due to the preservation of structure, function and/or gene expression should overall be manifest as negative or purifying selection. 

Patterns of selection in the mtORF of Pocilloporidae were investigated using the PAML package [99]. The overall nonsynonymous/synonymous substitution ratio (ω = dN/dS) as well as the relative rate of substitution between pairs of mtORF orthologs were calculated by constructing a maximum-likelihood tree, as described above, as well as a codon-based multiple-sequence alignment using the software pal2nal [100]. dN/dS was calculated using the program codeml with the following parameters: model = 0, Nsites = 0, icode = 4. 

In addition, the evolutionary models implemented in PAML were used to test site-specific variation in the mtORF evolutionary rate. Specifically, three likelihood ratio tests (LRTs) were carried out comparing first a neutral model allowing only ω = 0 and ω = 1 with uniform selective pressure among sites (M0) versus a model that allows variable selection among sites (M3), and second a model that assumes a beta distribution for ω allowing purifying selection (M7) versus a model that allows for positive selection (M8). Sites evolving under positive selection were identified using the Bayes Empirical Bayes approach [101]. By contrasting these models, the likelihood of identifying whether multiple classes of rates provide a better explanation of the data than a single class is improved.

#### 2.5.2. Codon Usage Bias 

In order to quantify codon usage bias in the mtORF, we calculated the codon adaptation index (CAI) [102]. The CAI quantifies the optimality of the codon composition of a gene by measuring the bias of its set of codons towards the codon frequencies in a reference table computed from a set of highly expressed genes. In the absence of gene expression data specific for mitochondrial genes in pocilloporids, this reference table was constructed for each species by simply counting the codons of all protein coding genes in their mitogenomes. This procedure relies on the assumption that most mitochondrial genes are optimally adapted for gene expression [103,104]. 

To assert whether the CAI was high enough to be considered biased toward codon optimality, we used a method to estimate the expected CAI (eCAI) [104]. This was done by simulating sequences of identical amino acid composition with respect to the query sequence, while accounting for the relative frequency of synonymous codons in the reference table. The resulting null distribution provides an upper confidence interval for significantly biased CAI. eCAI values reported here were calculated at the 99% confidence interval. Because circular genomes are known to have strong context-dependent nucleotide compositional biases, CAI analyses were also performed for mitochondrial genes located at neighboring genomic positions with respect to the mtORF.

### 2.6. Signatures of Adaptive Evolution in the mtORF of Stylophora Inhabiting Different Environments

**Figure 1:**
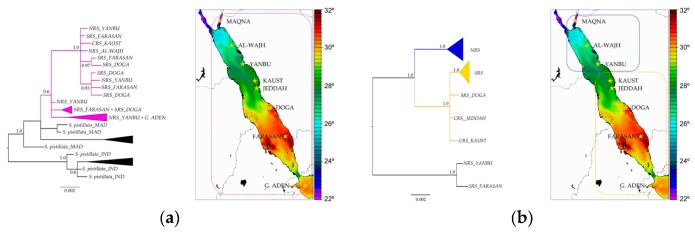
Phylogenetic position of Red Sea haplotypes and their distribution along the latitudinal and environmental gradients of the Red Sea. Graphs have been modified from those published by Banguera-Hinestroza et al. [15]. (**a**) Clade 1: *Stylophora RS_LinA* (magenta), *Stylophora pistillata* from Madagascar (MAD), and *Stylophora pistillata* from the Indo-Pacific region (IND). (**b**). Clade 2. *Stylophora RS_LinB*. Northern and southern populations are highlighted in blue and yellow respectively. Yellow stars indicate geographical locations. Haplotypes are named according to the sampling sites in which they showed the highest frequency (in the southern (SRS), northern (NRS), or central (CRS) Red Sea).

The potential involvement of the mtORF protein in local adaptation was tested by searching for signals of selection in the translated mtORF sequences. For this, mtORF haplotypes belonging to Red Sea *Stylophora* lineages as well as to *Stylophora* species from other oceanic regions were used. Samples included in these analyses (N = 827) belonged to the same set of samples analysed in Banguera-Hinestroza et al. [15], from which the definitions of *Stylophora* lineages, clades, subclades and populations were taken. Briefly, two *Stylophora* lineages were distinguished within two highly divergent clades: Clade 1 included lineage *RS_LinA* (~50% of *Stylophora* corals collected within the Red Sea and Gulf of Aden) and *Stylophora* specimens from the Indo-Pacific, Madagascar, Arabian Gulf and Gulf of Aden regions. Clade 2 included exclusively *Stylophora* specimens of the lineage *RS_LinB* (the remaining individuals from the Red Sea and Gulf of Aden areas). This clade was further divided into two subclades, each grouping specimens distributed either in the northern areas of the Red Sea or in the central-southern regions (Figure 1). For analyses of selection, all *Stylophora* sequences were first evaluated using regions that were unambiguously aligned among all haplotypes. In addition, sequences from Clade 1 (*RS_LinA* plus *Stylophora* from other oceanic basins) and Clade 2 (*RS_LinB*) were analysed separately. 

Last, selection was tested in sequences from *RS_LinA* and also in sequences from the northern and southern populations of *RS_LinB*.

Signatures of selection at individual sites on the mtORF-encoded protein were inferred using several approaches implemented in the Datamonkey webserver (www.datamonkey.org) [105,106]. (i) The Fixed Effects Likelihood method (FEL) detects selection by first estimating branch lengths and substitution rate parameters in a given phylogeny when the corresponding coding-region alignment is provided. After the calculation of these two parameters, the method works with fixed values to infer nonsynonymous (dN) and synonymous (dS) substitution rates per sites, allowing the identification of selection along the branches of the phylogenetic tree [107]. (ii) The Mixed Effects Model of Evolution (MEME) identifies episodic positive selection at individual sites [108] by combining both fixed models and random-effects models. (iii) The Branch-Site REL model (BS-REL) uses likelihood computations to infer the variation of dN and dS over branches and per site [109,110]. Statistical significance levels were set at a *p*-value of 0.1 for MEME and FEL and of 0.5 for BS-REL as recommended by the authors.

Furthermore, the M8 model of Yang et al. [111] and the Mechanistic–Empirical Combination (MEC) model from Doron-Faigenboim and Pupko [112] were applied to the mtORF protein using the Selecton webserver [113,114]. These approaches find the model that best fits the data by performing a likelihood ratio test (LRT) over the whole alignment. Here, a null model in which positive selection is not allowed (i.e., M8a; no-codons with dN/dS > 1) was compared against a general model that assumes positive selection (i.e., dN/dS > 1) as in the M8 and the MEC model. If positive selection was detected, a Bayes Empirical Bayes (BEB) approach was used to calculate the posterior probabilities that sites underwent positive selection [101]. Statistical significance was tested comparing the AIC (Akaike Information Content) scores between the MEC model or M8 model against the M8a model. Note that the likelihood ratio test was used to compare both models and the significance test was considered passed whenever the AIC score of M8 or MEC was lower than the M8a score using a threshold of 0.05. For details about the advantages of the MEC model, refer to Stern et al. [114].

The last test used to detect selection was the codon-based Z-test of positive selection of Nei and Gojobori [115] implemented in MEGA 7 [70]. This test allows discrimination between positive and purifying selection by performing pairwise comparisons of the relative rates of synonymous (dS) and non-synonymous substitutions (dN) and their variances [70] among all haplotypes, thereby enabling inferences of selection when specific clades or subclades are compared. The null hypothesis of dN = dS and the alternative hypotheses of dN > dS (positive/diversifying selection) and dN < dS (purifying selection) are tested using a one-tailed Z-test. Although this method is comparatively simple for testing selection hypotheses, it is considered to perform as well as more complex methods [116].

## 3. Results 

### 3.1. The mtORF Occurs in All Pocilloporids and Does Not Exhibit Stop Codons

**Figure 2:**
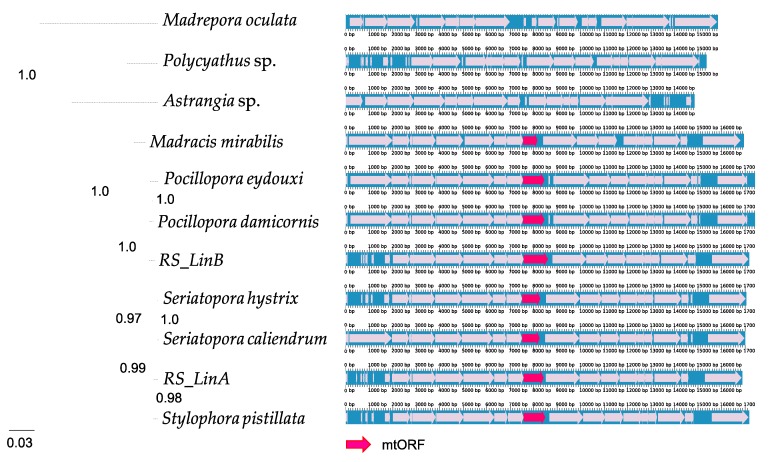
Family-level phylogeny showing that the mitochondrial Open Reading Frame (mtORF) likely appeared in the common ancestor of all pocilloporid corals. **Left**: maximum-likelihood tree based on a concatenation of most variable mtDNA genes (*nd2*, *nd6*, *atp6*, mtORF, and *nd4*). **Right**: full mitogenome alignments with the mtORF highlighted in red.

Phylogenetic analyses of the complete mitogenomes of corals within Pocilloporidae (genera *Madracis*, *Pocillopora*, *Seriatopora,* and *Stylophora*) in a phylogenetic framework and using three outgroups (*Madrepora*, *Polyciathus,* and *Astrangia*) showed that the mtORF likely appeared in the common ancestor of the family Pocilloporidae (Figure 2), which according to the calibrated coral phylogeny of Chuang et al. [68] probably existed 150–250 Mya. Stop codons were not found in the translated mtORF sequences of *Madracis*, *Pocillopora*, *Seriatopora,* and *Stylophora* (N = 1830), except in 3 out of 431 sequences from *Seriatopora* (accessions KR150027, KR150037, and KR150052 [13]; Supplementary Table S1). Examination of the chromatograms kindly provided by the authors showed that the apparent stop codons were the result of base calling errors, hence none of the 1830 sequences examined contains actually stop codons.

### 3.2. The mtORF-Encoded Proteins of Pocilloporids Vary in Length and in Aliphatic Indices

**Figure 3:**
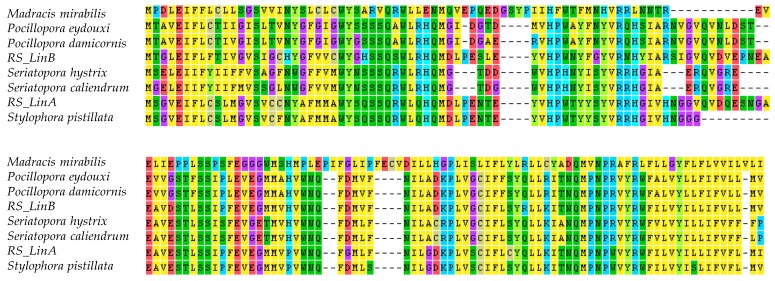
Conserved blocks in the mtORF-encoded protein of pocilloporid corals.

Translating the mtORF sequences of each genus resulted in proteins of different lengths. The shortest was found in *Madracis* with 221 amino acids and the longest was found in *RS_LinB* with 362 amino acids, followed by *Stylophora pistillata* with 309 amino acids, *Pocillopora* with 302, *Stylophora RS_LinA* with 301, *Seriatopora caliendrum* with 282, and *Seriatopora hystrix* with 269. The alignment of the conserved regions of the mtORF-encoded protein among genera is shown in Figure 3. Differences in length among and within *Seriatopora* and *Stylophora* sequences were found to be associated with the presence of duplicated and polymorphic tandem repeats (TRs) (Table 1) that generated long insertion-deletion (indels) in the alignments. In *Stylophora*, some of these TRs were exclusively found in sequences from Red Sea specimens of *RS_LinB* and motifs differed in length, number and/or nucleotide composition between southern and northern populations as well as among *Stylophora* lineages (Supplementary Table S2). 

Furthermore, the mtORF-encoded proteins for these genera differed also in their aliphatic indices, higher values of which indicate a higher thermostability [117]: calculated values were 112.4 for *Madracis*, 94.4 for *Pocillopora*, 88.3 for *RS_LinA*, 87.2 for *RS_LinB*, 85. 4 for *Stylophora pistillata* and 84.0 for *Seriatopora.*

### 3.3. mtORF-Encoded Proteins Contain Transmembrane Domains and Intrinsically Disordered Regions

**Figure 4:**
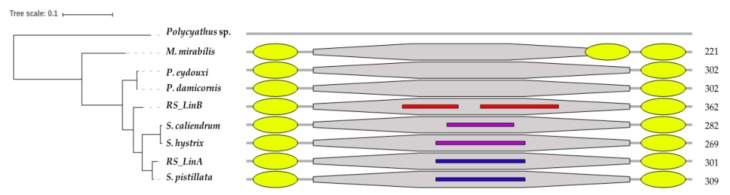
Domain organization. **Left**: maximum-likelihood phylogeny of the family Pocilloporidae based on the concatenation of *nd2*, *nd6*, *atp6*, *tmp362,* and *nd4.* **Right**: schematic representation of the TMP362 orthologs and their annotated domains. Sequences, domains, and IDRs differ in length, but for illustration purposes they are shown aligned. Yellow ellipses represent transmembrane domains (TMDs); grey octagons, hydrolase domain (HDs); thin rectangles, disordered regions found as the consensus of several methods (red) or only reported by DISOPRED (blue), or InterproScan and IUPred2A (magenta). Numbers on the right-hand side indicate the length of each protein.

Exploration of domain architecture using five predictors of secondary structure and transmembrane domains (TMDs) suggested that the mtORF encodes a transmembrane protein. From this point on we shall refer to this protein as TMP362 (short for TransMembrane Protein 362 amino acids long, as in the case of *RS_LinB*) and to the corresponding gene as *tmp362*. In *Madracis*, TMP362 comprises three well-supported TMDs (one at the N-terminal side and two at the C-terminal side), versus two (one at each side) in the other pocilloporids genera (Figure 4 and Supplementary Figure S1). Often signal peptides can be misannotated as TMDs, but there was no evidence for signal peptides in TMP362 using MEMSAT or signaIP, suggesting that our predictions indeed correspond to TMDs. These annotations are not surprising since most mitochondrial protein-coding genes are associated to the mitochondrial membrane. A third TMD was predicted with high probability at the C-terminal in *Seriatopora* by the MEMSAT3 approach but was not supported by the other methods. If we assume that this third TMD really exists, it suggests that TMP362 comprised three TMDs in the common ancestor of all pocilloporids.

**Figure 5:**
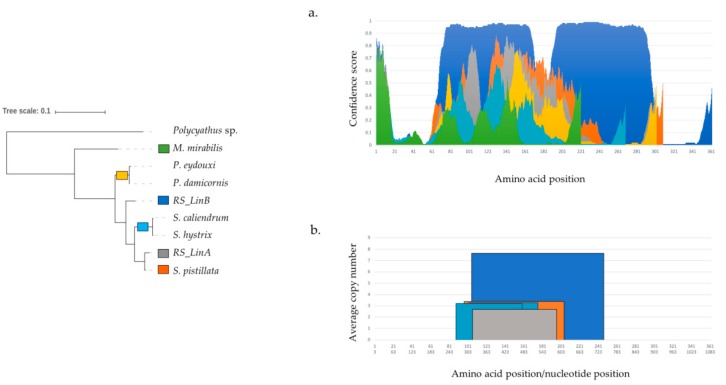
Intrinsic disorder profile and tandem repeats. (**a**). Intrinsically disordered regions in pocilloporid corals predicted by DISOPRED3. Amino acids with a confidence score above 0.5 are considered disordered, predicted at a false positive rate threshold of 0.05 [83,85]. (**b**). Tandem repeats in representative sequences of *tmp362* of *Stylophora* and *Seriatopora* species.

In addition, analyses revealed intrinsically disordered regions (IDRs) in TMP362 (Figure 5). In *RS_LinB* IDRs span 216 amino acids, from residue 68 to 173 and from residue 188 to 297. Results from DISOPRED, which is specifically trained to identify disordered regions by their intrinsic characteristics [118], indicated that IDRs are not fully conserved among *Stylophora* lineages. In *Stylophora pistillata*, 79 amino acids were predicted as IDRs (from residue 123 to 201), whereas in the protein of *RS_LinA* IDRs cover two small regions with a total of 27 amino acids, from residue 134 to 153 and from 160 to 166 (Figure 5a). These residues were undetectable by other predictors centered on sequence information, such as IUPred. This predictor is based on a pairwise energy-like parameter derived from sequences in globular proteins [85] and suggested disordered regions in *Seriatopora* and *RS_LinB* but not in other *Stylophora* lineages (Figure 4). Noticeably, in all species the predicted IDRs coincide with a region rich in duplicated and polymorphic TRs (Table 1, Figure 5b, Supplementary Table S2). 

### 3.4. Remote Homologs Suggest that mtORF has a Hydrolase Domain

Despite the presence of disordered regions, a large fraction of internal domain of TMP362 seems to be structured. The FFAS method detected a far, although significant homology with a known structure from the Protein Data Base (PDB ID: 3b57, chain A) that corresponds to a bacterial protein known as lin9899, not characterized experimentally till now but with a hydrolase (HD) domain annotated based on homology. The HD domain belongs to the all-α structural class that is highly conserved across Bacteria, Archaea, and Eukarya and is involved in nucleic acid metabolism and signaling [119,120]. This domain is often associated to a metal-dependent phosphohydrolase function (EC 3.1). 

Interestingly, the HD domain covers a large fraction of the internal domain of the TMP362, spanning approximately 100 residues (residues 25 to 132 in 3b57, chain A) close to the TMP362 C-terminal end (a schematic view is presented in Figure 4). The canonical catalytic site in the HD domain involves histidine (H) and aspartic (D) residues responsible for metal binding. A multiple sequence alignment with the homolog of the known structure (3b57) suggests that a fully conserved histidine (H, at position 23 with respect to 3b57), as well as several relatively conserved aspartic residues (D, e.g., at position 104 with respect to 3b57), might still be involved in a hydrolase activity or might be the relict of a former HD functional domain. The finding of a HD domain homolog is supported by annotations carried out using the Gene Ontology Annotation system (GOA), which found GO terms uncovering three molecular functions: catalytic activity (0.53 < P < 0.77), hydrolase activity acting on acid anhydrides (0.56 < P < 0.92), and cytoskeletal protein binding activity (0.59 < P < 0.68). Furthermore, the catalytic function of a hydrolase was supported with largely significant posterior probabilities (P > 0.75) (Table 2).

### 3.5. Biological Processes and Cellular Localization Support the Hypothesis of a Mitochondrial Transmembrane Protein 

Two main biological processes were suggested by GO terms for the role of TMP362 in all the pocilloporid genera and lineages examined (Table 3): transport (0.80 < P < 0.87) and regulation of metabolic processes (0.71 < P < 0.85), except in *RS_LinB* where the cell surface receptor signaling pathway had the highest posterior probability after transport (P = 0.79). Other processes were predicted with moderated probabilities and two were only suggested for the TMP362 of *Madracis* and *Seriatopora* (i.e., ion transmembrane transport and nucleotide metabolic process; Table 3). Furthermore, predictions for cellular localization yielded a high probability and reliability for this protein being an intrinsic and integral component of the membrane (0.97 < P < 1.00) in all genera, with moderate to high probabilities of being part of an organelle membrane (i.e., mitochondria; 0.70 < P < 0.80). Overall, these analyses support the hypothesis that TMP362 is a mitochondrial transmembrane protein with possible transport, signaling and/or metabolic functions.

### 3.6. Strong Signatures for Positive and Negative Selection are Detected in the mtORF of Pocilloporid Corals

If the mtORF encodes a functional protein, then some form of selection for the preservation of expression and/or function should be at play. In order to test this hypothesis, an evolutionary rate analysis of the *tmp362* gene across species was carried out. First, the evolutionary rate of pairs of *tmp362* orthologs (i.e., from the 8 species of pocilloporids included here) was evaluated under the assumption that there was a single average rate along the sequence. This analysis revealed that on average *tmp362* has experienced negative selection (i.e., dN/dS < 1.0). For instance, with respect to *Madracis*, *Pocillopora* is on average evolving at a dN/dS = 0.53, *Seriatopora* at 0.44, and *RS_LinB* and *RS_LinA* at 0.43 and 0.6, respectively. The assumption of a single rate along an entire gene often averages out signatures of selection operating in few sites along the sequence. Thus, it is possible that while some sites are evolving under strong negative selection, some are positively selected (dN/dS > 1.0) or experience neutral evolution (i.e., dN/dS= 1.0). To explore these alternatives, a likelihood ratio test (LRT) was implemented. This test contrasts a model that assumes a single constant rate (M0), versus a model that allows for a set of discrete classes of sites (M3). By comparing these two models, it can be asserted whether multiple classes of rates do indeed provide a better explanation of the data than a single class. Here, the LRT provided strong evidence in favor of the M3 model, suggesting that distinct groups of sites in *tmp362* are evolving at considerably different rates (M3 & M0: 2Delta*l* = 95.48; df = 4, *p*-value < 0.001). 

In addition, a comparison of M3 models using an increasing number of rate classes provided significant support for three versus two, but not for four versus three classes (M3 (K = 3 and K = 2): 2Delta*l* = 7.95; df = 2, *p*-value < 0.05). This analysis showed that 36% of sites are under strong purifying selection (dN/dS = 0.1), whereas 18% are experiencing positive selection (dN/dS = 4.3). The remaining 46% are evolving in a relatively neutral way. A Bayes Empirical Bayes approach indicated that four of the positively selected sites (i.e., 27A, 34I, 47V and 65V, all of which located close to the N-terminal end of the protein) are associated to high posterior probabilities (P > 0.97). 

A codon bias analysis revealed that the codon usage in the *tmp362* of all pocilloporid coral species examined in this work is significantly biased with 99% confidence. The eCAI analysis gave evidence supporting that mitochondrial genes under similar context-dependent mutations are subject to similarly significant biases in codon usage as *tmp362.* This suggests that *tmp362* is fully functional and not a pseudogene, in accordance with the results of subsequent analyses (see below). 

### 3.7. Stylophora Corals in the Red Sea Exhibit Signatures of Selection in the Their mtORF-Encoded Protein Along a Latitudinal Gradient

To test whether *tmp362* might play a role in adaptation to the environment, the hypothesis of selection was tested on populations of *Stylophora* corals adapted to a range of different environmental conditions in the Red Sea. *Stylophora* specimens from other regions were included for comparison. Amino acid sites under positive/diversifying selection at two sites were identified with the MEME approach when sequences from Clade 1 –*RS_LinA* and *Stylophora pistillata* (i.e., specimens from Pacific and Indian Oceans, including Madagascar)– were evaluated together. Positive selection (at 5 sites) was also found when the analyses were restricted to Red Sea sequences from *RS_LinA* and *RS_LinB* (the phylogenetic position and distribution of these lineages are illustrated in Figure 1). However, positive selection was not detected when the alignment included the small section of TMP362 aligned among all *Stylophora* sequences (evaluating only unambiguously aligned regions) or when sequences from each *Stylophora* lineage were analysed separately. 

**Figure 6:**
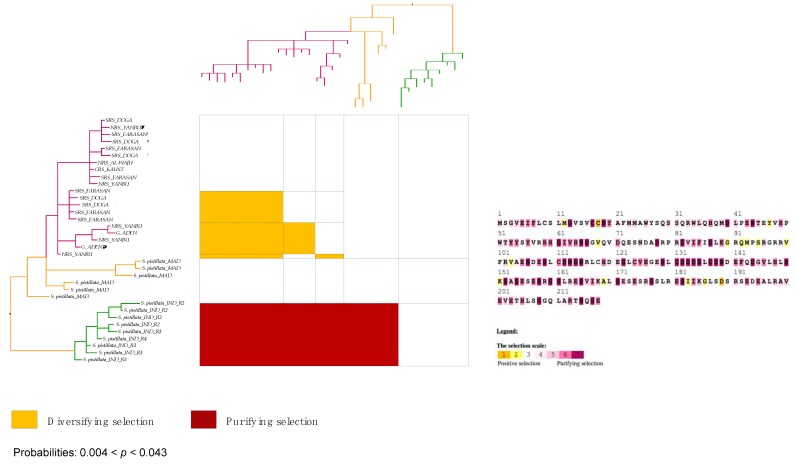
**Left**: graphical view of the codon-based test of purifying and positive/diversifying selection (Nei and Gojobori [115]) in pairwise comparisons between haplotypes of *Stylophora* within Clade 1 (*RS_LinA* plus Indian and Pacific Oceans samples). **Right**: sites under positive and purifying selection predicted by the MEC approach. The phylogenetic tree has been modified from Banguera-Hinestroza et al. [15]. For simplicity, haplotypes have been named according to their region of origin (see Figure 1).

**Figure 7:**
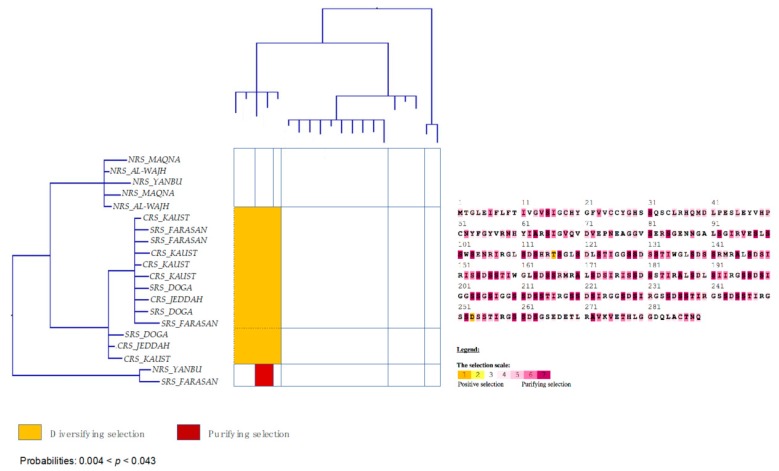
**Left**: graphical view of the codon-based test of purifying and positive/diversifying selection (Nei and Gojobori [115]) in pairwise comparisons between haplotypes of *Stylophora* within Clade 2 (*RS_LinB*). **Right**: sites under positive and purifying selection predicted by the MEC approach. The phylogenetic tree has been modified from Banguera-Hinestroza et al. [15]. For simplicity, haplotypes have been named according to their region of origin (see Figure 1).

The M8 and MEC model recovered the highest number of single amino acid sites under positive selection in most cases (Figure 6 and Figure 7). Nevertheless, only the MEC model showed significant values for most comparisons, namely: (*i*) in the TMP362 of *Stylophora RS_LinA* and *Stylophora pistillata* at one site (the AIC score for MEC: 2846.897 was lower than for M8a: 2852.35), (*ii*) when the alignment included sequences from both Red Sea lineages (*RS_LinA* and *RS_LinB*; AIC score for MEC: 1468.55; AIC score for M8a: 1472.37), and (*iii)* when sequences from all *Stylophora* specimens were analysed (i.e., MEC score = 1737.074; M8a score = 1745.86).

Analyses of sequences from the southern and northern group of *RS_LinB* independently indicated sites under positive selection in the TMP362 of the southern group (AIC score for MEC: 2313.488, AIC score for M8a: 2317.705). This was corroborated by the results of the Nei-Gojobori method with highly significant *p*-values (0.003 < *p* < 0.043, Figure 6). Furthermore, positively selected sites were not found in the northern group of this lineage nor in sequences of *RS_LinA* alone (except in the TMP362 of specimens whose haplotypes had the highest frequencies in the southern Red Sea or were restricted to YANBU, a sampling site found at the boundary between northern and central Red Sea oceanic provinces; Figure 1). A graphical view of positively and negatively selected sites using the MEC approach as well as a graphical summary for the outputs of the Nei-Gojobori method are included in Figure 6 and Figure 7 (the full matrices are available upon request). 

Congruent with the finding in Section 3.6, amino acid sites under pervasive negative/purifying selection were found in all alignments of TMP362 using the FEL method: at 11 sites for all *Stylophora* lineages; at 7 sites for *Stylophora pistillata* and *RS_LinA*, at 6 sites for *RS_LinB* and also at 6 sites when *RS_LinA* and *RS_LinB* sequences were placed together. When the southern and northern group of *RS_LinB* were analyzed separately, one site was found under purifying selection in the TMP362 of each group. Furthermore, purifying selection was also detected in mtORF-encoded protein of *Stylophora pistillata* using the Nei-Gojobori method (0.003 < *p* < 0.043). BS-REL did not detect sites under selection along the branches of the phylogenetic tree of this genus.

## 4. Discussion

The mitochondrial genome of plants and animals have been shown to be prone to loss and/or acquisition of new genes [7,121,122]. Some of these genes do not show homology with proteins of known function, but are fully functional [1,4,7]. In this work, we carried out a computational characterization of a putative protein-coding gene previously found in the mitochondrial genome of corals of the family Pocilloporidae. To test the hypothesis that the mtORF of pocilloporids is a functional gene, we examined single-gene and whole mitochondrial genome alignments, ran several predictors of protein features, performed structural and functional annotations, looked for signatures of selection, and investigated codon-usage biases. Particularly, we focused on previously reported *Stylophora* lineages [15] evolving along the full latitudinal and temperature gradients of the Red Sea, one of the hottest marine basins on Earth that experiences southern temperatures above 30 °C [43,44,123], the thermal limit for corals in most parts of the world [124].

Here we discuss our analyses broadly, first in the context of protein structure and function and second in light of the current knowledge on the role of structural changes in adaptation. In this regard, we explore whether the patterns of nucleotide substitutions and structural changes in the mtORF-encoded protein dubbed here TMP362 might be explained by the current knowledge of the role of mitochondrial proteins, tandem repeats (TRs) and intrinsically disordered regions (IDRs) in adaptation. This, we hope, will inform the current efforts to understand the signatures of environmental pressures on the mitochondrial genome of marine species, particularly on reef-building corals. 

### 4.1. Origin and Conservation of tmp362 

Our analyses present substantial evidence suggesting that the mtORF gene *tmp362* arose in the most recent common ancestor of the family *Pocilloporidae* around 150–250 Mya. The consensus of several prediction methods revealed that *tmp362* encodes a transmembrane protein in all genera studied here, in agreement with its preliminary characterization by Flot and Tillier [9]. Overall, the competing hypothesis that *tmp362* is a pseudogene was not supported by our analyses. Indeed, an extensive analysis including 1830 partial and complete sequences of this gene, representative of several Pocilloporidae species, did not show a single stop codon in the translated protein. Furthermore, comparisons of evolutionary models revealed pervasive purifying selection, several sites under positive selection, and a bias in codon usage comparable to or even stronger than that showed by other mitochondrial genes in neighboring genomic regions. 

### 4.2. Putative Homology with a Bacterial Hydrolase Domain

Homology searches revealed the existence of a handful of significant far homologs of known structure. The top-ranking homologs correspond to bacterial proteins annotated with a hydrolase domain, which spans the internal region of TMP362. However, homology detection at low levels of significance is error-prone, hence this result should be interpreted with caution. First, although the hydrolase homology was detected in all mtORF orthologs when using a multi-alignment strategy, it was not detected in all pocilloporid mtORF sequences when analyzing them separately. Second, some predictors suggested the presence of β-sheets, but the hydrolase domain present in the far homolog is associated to an all-α fold. 

Multiple disordered regions associated to tandem repeats (see discussion below), as predicted by our analyses in *Stylophora* and likely in *Seriatopora*, might have introduced several structural changes into the ancestral domain, and probably also explain the variations in secondary structure among genera. Although this suggests that the hydrolase domain might not be fully conserved among pocilloporids, an independent analysis of functional annotation using Gene Ontology also predicted a hydrolase catalytic function with highly significant posterior probabilities for all TMP362 orthologs studied here (Table 2 and Table 3). This additional evidence supporting structural annotation, plus the fact that reef-building corals are holobionts harboring multiple symbiotic organisms including photosynthetic algae, viruses, and bacteria [125,126], lead us to propose that *tmp362* is of bacterial origin and was horizontally transferred to the mitochondrial genome of the common ancestor of all pocilloporid corals. Mutation accumulation mainly in form of tandem repeats, intragenomic recombination (as suggested by Banguera-Hinestroza [15]), and possibly, adaptation to varying environmental conditions, resulted in strong genotypic variation. Despite the nucleotide-level and structural diversity of *tmp362* among pocilloporid corals, its encoded protein remains apparently functional in all the genera and lineages examined. 

### 4.3. Putative Role of tmp362 in Environmental Adaptation

#### 4.3.1. Tandem Repeats (TRs) and Intrinsically Disordered Regions (IDRs)

TRs were the main source of variation among sequences of *tmp362* in most pocilloporids. Noticeably, the highest number of repeat units was found in the lineage endemic to the Red Sea (*RS_LinB*), in which copy number varies between 3 and 17 (Table 1). TRs were also found, although to a lesser extent, in *Seriatopora* and *Stylophora* lineages with broader distribution: in *RS_LinA* (likely restricted to the Red Sea, Arabian Gulf, Gulf of Oman, and Gulf of Aden) and *Stylophora pistillata*, *S. caliendrum*, and *S. hystrix* (widely distributed in the Indo-Pacific region). Refer to [15] and [41,127] for the distribution of Red Sea and Indo-Pacific *Stylophora* lineages respectively. Moreover, in pocilloporids, TRs were associated with predicted IDRs in TMP362, except in *Pocillopora* and *Madracis* in which neither TRs nor IDRs were found (Figure 5).

Analysis of a large number of *tmp362* sequences (N = 827) from Banguera-Hinestroza et al. [15] revealed differences in number, length, and nucleotide composition of repeat motifs among *Stylophora* lineages inhabiting distinct oceanic basins. Conversely, indels and TRs motifs in *tmp362* sequences were found to differentiate northern and southern populations of *RS_LinB* (restricted to the coldest and hottest areas of the Red Sea, respectively; Figure 1, Supplementary Table S2).

Interestingly, indels and TRs have been found to be associated with patterns of evolution, ecological diversification, and adaptation in a range of marine and terrestrial organisms, including viruses [128,129], bacteria [130] (reviewed in [131,132]), plants [133,134,135], and animals [136,137,138]. Indeed, the genome of organisms evolving under strong selective pressures often exhibit a proliferation of TRs (reviewed in [139]), which has been explained as the results of rapid adaptive changes [140,141,142] arisen in response to the environmental stress imposed by the colonization of new environments, drastic environmental changes, or global warming [132,134,139,143,144]. TRs are also involved in protein function [139,145], increase functional variability [146] and play key roles in the regulation of gene expression in organisms growing under different stressors [132,147]. In humans, for example, mutations that affect TR number have been found to increase the fitness of the cell when exposed to stressful conditions (e.g., cold, heat, hypoxia, oxidative stress) by adjusting the regulatory network that enhances protein activity and gene expression [148].

On the other hand, IDRs as predicted here for TMP362 of *Stylophora* and likely *Seriatopora* lineages, (Figure 5) have been found to be a common feature of the genome of eukaryotes (reviewed in [84,149,150]). These regions evolve mostly through the expansion of functional TRs (reviewed in [151]) and indels [152], and their degree of polymorphism has been hypothesized to be the result of the strength of evolutionary forces leading to adaptive changes [153,154]. Furthermore, IDRs confer functional advantages to a protein, providing the adaptability that allows them to bind to different partners (i.e., functional promiscuity) with different regions (domains) often found associated to different functions [149,155,156]. In fact, the functional promiscuity as well as the conformational diversity found in some enzymes allow them to evolve new activities rapidly [153]. 

IDRs appear to be highly represented in proteins of organisms evolving in extreme environments (i.e., prokaryotes [157]). Their role in response to abiotic stress have been mainly recorded in plants [158,159]. However, it has been shown that they perform important functions to protect the cell from thermal stress in other organisms, including cold conditions in bacteria [160] and high temperatures in animals (e.g., under desiccation conditions in tardigrades [161]). This is in agreement with early studies showing that flexibility is a key characteristic of proteins involved in thermal adaptation (reviewed in [162]). 

On the other hand, there is a mounting evidence showing that similar to TRs, IDRs are essential for a diversity of cellular processes (see review in [163]), some of them key for adaptation to the environment. For instance, these regions facilitate macromolecular interactions by participating actively in the assembly of signaling complexes, interacting with structured domains in other proteins [84,163,164]. This is particularly relevant in mitochondrial proteins that interact closely with their nuclear partners in mito-nuclear interactions and have been found to be key players not only in adaptive responses but also in speciation [27,33,35]. 

Noticeably, the main processes predicted for biological function of the TMP326 of *RS_LinB* (i.e., transport and cell surface receptor signaling pathway [P = 0.8]) were similar to those processes found in disordered proteins of other metazoans, the IDRs of which have been associated with regulation of cellular processes, including intracellular signaling, membrane fusion and transport, and signal transduction [84,164]. However, these results should be taken with caution as a fully experimental characterization of this protein will be needed to unravel its function in the mitochondrial genomes of pocilloporids, particularly in species evolving in environments considered extreme for reef-building corals. 

#### 4.3.2. Structural Diversification of TMP362 

TMP362 presents three well-supported transmembrane domains in *Madracis* versus two in the other pocilloporids (but a third transmembrane domain may be present in *Seriatopora*; Supplementary Figure S1). Interestingly, differences in the number of TMDs between homologous proteins have been recorded in plants and others photosynthetic organisms. For example, changes in amino acid sequences leading to differences in secondary structures have been reported in two rice lines (i.e., Huhan1A and Huhan 1B) that differ in their tolerance to stress [165]. In an experimental study, authors found a reduction of TMDs in the proteins encoded by the mitochondrial genes *ccmB* and *ccmC* of Huhan1A and Huhan 1B, from 5 to 3 and 6 to 4 respectively, when analyzing gene expression under different environmental settings. These differences were interpreted as a consequence of deficiencies in RNA editing due to oxidative stress, which is also associated to other stressors such as drought, heat and cold [166]. 

At a broader evolutionary scale, changes in TMDs have been also reported in homologous proteins of the light-harvesting complex (LHC)-like family, which exhibits a broad functional diversity and has acquired novel functions throughout the evolution of photosynthetic organisms [167,168]. During evolution, these proteins have gained and lost TMDs multiple times. The ancestral protein likely evolved from one to two TMDs and acquired two extra TMDs by duplication, with one domain loss (from 4 to 3) in the homologous protein of extant lineages (e.g., cyanobacteria and photosynthetic eukaryotes; reviewed in [169,170]). Remarkably, LHC homologs are transmembrane proteins involved in photo-protective responses, protecting against the photo-oxidative damage caused by excessive production of reactive oxygen species, particularly in periods of long exposure to sunlight [171,172,173]. They are also induced as a response to other environmental stressors such as increases in salinity and temperature, and during drastic decreases in nutrients availability (reviewed in [174]).

Retracing the more likely path for the evolutionary changes in the structure of TMP326 orthologs is out of the scope of the present work. However, based on the studies outlined here, a possible explanation could be that the structural differences among TMP326 orthologs evolved as a response to different adaptive pressures, possibly related to environmental changes. Studies in reef-building corals have shown that the response to environmental stressors, such as heat stress, may differ among taxa [175]. In some corals, however, this response is under a strong maternal influence and is characterized by the overexpression of set of mitochondrial proteins [176]. Such studies are still lacking for reef-building corals and will require experimental characterizations of mtDNA-encoded proteins involved in stress response to understand their significance in an evolutionary context. 

#### 4.3.3. Signatures of Selection in the TMP362 of Stylophora Lineages along Latitudinal and Environmental Gradients

A third line of evidence in support of the hypothesis of a role of the TMP362 protein in adaptation is the signal of positive selection in sequences of specimens inhabiting the warmest regions of the Red Sea, particularly in sequences from *RS_LinB* (Figure 6 and Figure 7), versus an absence of positively selected sites in TMP362 of specimens restricted to the coldest northern areas. Noticeably, TMP362 of individuals from both lineages, distributed along the full environmental gradient of the Red Sea, showed multiples sites under purifying selection (Figure 6 and Figure 7). This finding was supported by all methods (see results Section 3.7 and Figure 1 for reference to the geographic distribution of these lineages in the Red Sea).

It is broadly accepted that coral reef ecosystems accompanied the evolution of the Red Sea basin since its incipient stage in the early Miocene [177], and that pocilloporid corals likely entered the region as early as the Pliocene-Pleistocene [177,178,179,180,181]. During these periods they faced a variety of environmental stressors, such as extended periods of hyper-salinity, changes in nutrient availability, and strong fluctuations in oceanic temperatures and sea levels [50,182,183] that lead to the extinction of some lineages, while others survived in refugia in the northern and southern Red Sea (reviewed in DiBattista et al. [48]). These environmental settings might explain the signals of adaptive evolution as well as the unique characteristics of the mtORF-encoded protein of the endemic lineage *RS_LinB*, which may have experienced more extreme fluctuations in environmental conditions than corals evolving under less stressful conditions. 

On the other hand, the lack of positive selection in the mtORF-encoded protein of *RS_LinA* contrasts with the multiple amino acid sites found to be under purifying selection in this lineage. This could be explained by its relatively recent appearance in the Red Sea, in agreement with its hybrid origin [15]. In fact, more recent *Stylophora* lineages likely entered the Red Sea within the last 4–7k years, when environmental conditions were similar to those recorded nowadays [184]. They overcame the bottleneck of high temperature waters in the southern Red Sea (up to 34 °C) and then spread north along with other coral species [185]. 

Overall, these findings are congruent with results based on protein structure and function, discussed in the previous sections, suggesting that the mtORF-encoded protein may indeed have a role in environmental adaptation, likely to high temperatures, highlighting also its functional importance. However, as already mentioned, this hypothesis will require further testing using experimental data and a combination of proteomics, genomics and ecological approaches.

## 5. Conclusions

Our study not only offers insights into the coding nature of the mtORF gene in pocilloporid corals, but adds to our knowledge of the main characteristics of the mtORF-encoded protein. Striking dissimilarities were found among the TMP362 proteins of pocilloporid corals, with the greatest differences found among *Stylophora* lineages, including changes in structure. The most divergent protein was that of *RS_LinB*, a lineage that was hypothesized to have adapted to the extreme environments of the Red Sea during an early colonization, likely predating that of other *Stylophora* lineages. Several features found in the TMP362 of the *RS_LinB* (i.e., high frequency of TRs, long stretches of disordered residues, as well as signals of selection) show trends similar to proteins of organisms surviving under severe conditions and/or exposed to damage by oxidative stress, as most reef-building corals under thermal stress. Therefore, our findings suggest that *tmp362* and likely other genes involved in mitochondrial interactions have played a key role in the adaptation of *Stylophora* corals to extreme environments and fluctuating conditions.

This study, therefore, opens the door for further studies looking at the role of mitochondria and mitochondrial genes in the adaptation of coral species during their diversification. Corals and coral reefs are severely threatened by climate change (e.g., sea surface temperature rise), hence, more detailed studies on coral adaptive mechanisms are required to understand the potential responses of corals in both ecological and evolutionary scales. We consider the understanding of protein–protein interactions at the mitochondrial level of particular relevance, since they appear to play a key role in coral adaptation to strong environmental changes.

## Figures and Tables

**Table 1:** Tandem repeats in the mtORF sequences of representative lineages of *Stylophora* and *Seriatopora* corals.

	Indices	Period Size	Copy Number	Consensus Size	Percent Indels
*RS_LinB*	315–741	27	16.9	27	11
	318–741	54	8.4	54	13
	371–613	75	3.3	75	5
	501–634	21	6.1	21	10
	486–741	69	3.5	69	9
*S. hystrix*	310–487	51	3.5	51	0
	352–430	24	3.2	24	10
	310–528	51	4.3	51	1
	306–525	102	2.2	102	1
*S. caliendrum*	265–436	51	3.4	51	0
	265–477	102	2.1	102	1
	265–477	51	4.2	51	1
*RS_LinA*	317–424	39	2.6	42	4
	365–440	21	3.8	21	10
	350–444	39	2.4	39	7
	480–587	51	2.1	51	0
*S. pistillata*	290–417	39	3.2	42	6
	338–413	21	3.8	21	10
	453–611	51	3.1	51	0

**Table 2:** Predicted molecular functions for TMP362 of pocilloporid corals.

		Posterior Probabilities					
GO Term	Molecular Function	*Madracis*	*Pocillopora*	*Seriatopora*	*Stylophora RS_LinB*	*Stylophora RS_LinA*	*Stylophora pistillata*
							
GO:0005216	ion channel activity	0.913	-	0.628	-	-	0.581
GO:0016817	**hydrolase activity, acting on acid anhydrides**	**0.902**	**0.862**	**0.561**	**0.824**	**0.695**	**0.626**
GO:0015075	ion transmembrane transporter activity	0.88	-	0.654	-	-	-
GO:0022890	inorganic cation transmembrane transporter activity	0.864	-	-	-	-	-
GO:0008324	cation transmembrane transporter activity	0.858	-	-	-	-	-
GO:0005524	ATP binding	0.833	-	0.832	-	0.739	0.502
GO:0046873	metal ion transmembrane transporter activity	0.787	-	-	-	-	-
GO:0015077	monovalent inorganic cation transmembrane transporter activity	0.749	-	-	-	-	-
GO:0003824	**catalytic activity**	**0.748**	**0.762**	**0.771**	**0.528**	**0.739**	**0.681**
GO:0016818	**hydrolase activity, acting on acid anhydrides, in phosphorus-containing anhydrides**	**0.707**	**0.602**	**0.602**	**0.666**	**0.695**	**0.619**
GO:0022857	transmembrane transporter activity	0.706	0.56	0.696	-	-	0.502
GO:0005261	cation channel activity	0.688	-	-	-	-	-
GO:0005215	transporter activity	0.67	0.548	0.684	-	0.561	0.515
GO:0035639	purine ribonucleoside triphosphate binding	0.639	0.538	0.706	-	0.733	0.538
GO:0008092	**cytoskeletal protein binding**	**0.588**	**0.592**	**0.598**	**0.682**	**0.62**	**0.676**
GO:0000166	nucleotide binding	-	0.52	0.71	-	0.72	-
GO:0001882	nucleoside binding	-	-	0.69	-	0.752	-
GO:0032549	ribonucleoside binding	-	-	0.682	-	0.779	-
GO:0017076	purine nucleotide binding	-	-	0.604	-	0.779	0.557
GO:0022891	substrate-specific transmembrane transporter activity	-	-	0.68	-	-	-
GO:0030554	adenyl nucleotide binding	-	-	0.648	-	0.547	-
GO:0016301	kinase activity	-	0.53	0.549	0.617	0.857	0.655

**Table 3:** Predicted biological processes for TMP362 of pocilloporid corals.

		Posterior Probabilities					
GO Term	Biological Process	*Madracis*	*Pocillopora*	*Seriatopora*	*Stylophora RS_LinB*	*Stylophora RS_LinA*	*Stylophora pistillata*
							
GO:0006810	**transport**	**0.862**	**0.847**	**0.874**	**0.803**	**0.824**	**0.82**
GO:0034220	ion transmembrane transport	0.845	-	0.689	-	-	-
GO:0019222	**regulation of metabolic process **	**0.813**	**0.853**	**0.795**	**0.711**	**0.849**	**0.791**
GO:0007166	**cell surface receptor signaling pathway**	**0.804**	**0.63**	**0.564**	**0.79**	**0.662**	**0.717**
GO:0009117	nucleotide metabolic process	0.728	-	0.697	-	-	-
GO:0051649	**establishment of localization in cell**	**0.694**	**0.692**	**0.721**	**0.538**	**0.698**	**0.659**
GO:0051641	**cellular localization**	**0.647**	**0.631**	**0.64**	**0.632**	**0.643**	**0.636**

## Supplementary Materials

The following are available online at https://www.mdpi.com/2073-4425/10/5/324/s1, Figure S1: Transmembrane domains and pore-lining regions in pocilloporid corals; Table S1: Accession numbers of mtORF sequences analyzed in this study; Table S2: TRs motifs in Stylophora lineages and populations; Data S1: Full mitogenomes of RS_LinA and RS_LinB; Data S2: Translated mtORF sequences of Pocilloporidae corals.
