# Supplementary material for: Computational Characterization of the mtORF of Pocilloporid Corals: Insights into Protein Structure and Function in Stylophora Lineages from Contrasting Environments
Source: Genes (Basel). 2019 Apr 27;10(5):324. doi: 10.3390/genes10050324 (PMC6562464; doi:10.3390/genes10050324)
Supplement: Supplementary file 1 [file genes-10-00324-s001.zip › Supplementary_26042019/Supplementary _Table_S2.pdf]

Supplementary Table S2. TRs motifs in S*tylophora* lineages and populations

**CLADE 1**<br>**(*Stylophora RS_LinA* plus *Stylophora* Indo-Pacific)**	**Consensus sequence**	**Period size**	**Copy number**	**Consensus size**	**Percent indels**	**A**	**C**	**G**	**T**
***RS_LinA*, Red Sea**	AAGTGAGTCAGAAAGTAGAGGTAGTTTGAGGGAAAGTATAATTAAGGCATT	51	2.1	51	0	38	2	33	25
	AAGTGAGTCAGAAAGTAGAGGTAGTTTGAGGGAAAGTATAATTAAGGGATT	51	4.1	51	0	39	2	32	25
	ATGACGAGAGTTTGTGTGATAATGATAATCGTCTGTGTGTTC	39	2.6	42	4	22	7	33	37
	TGTGTCATGACGAGAGTTTG	21	3.7	20	12	20	6	36	36
	GTTTGTGTGATAATGATGATA	18-21	2.8-3.8	21	10-16	21-23	1-3	30-32	42-43
	AATGACGAGAGTTTGTGTGAT	18	2.4	21	13	29	4	33	33
	AGTTTGTGTGATAATGAT	18	1.9	18	0	25	0	31	42
**Madagascar**	AAGTGAGTCAGAAGGTAGAGGTAGTTTGAGGGAAAGTATAATTAAGGCATT	51	2.1	51	0	38	2-3	33	24-25
	TGTGTCATGACGAGAGTTTGTGTGAATGATGATAGTTTG	39	2.4	39	7	21	6	34	37
	ATGACGAGAGTTTGTGTGATAATGATAATCGCTTGAGTC	39	2.7	39	4	23	7	33	36
**Pacific Ocean**	AAGTGAGTCAGAAAGTAGAGGTAGTTTGAGGGAAAGTGTAATTAAGGGATT	51	2.1	51	0	37	1	35	25
	ATGACGAGAGTTTGTGTGATAATGATAATCGTTTGTGTGTTC	39	3.2	42	6	22	6	33	37
	ATGACGAGAGTTTGTGTGATAATGATAATCGTTTGTGTC	39	2.6	39	0	24	4	32	38
	GTTTGTGTGATAATGGATC	18	4.8	19	12	21	4	32	41

**CLADE 2**<br>**(*RS_LinB*)**	**Consensus sequence**	**Period size**	**Copy number**	**Consensus size**	**Percent indels**	**A**	**C**	**G**	**T**
**Northern and southern Red Sea**	AATTAGGGGATCAAGTGATTC	21	6-11	21	10	32-34	9	29-31	26
	CAATTAGGGGATCAAGTGATTCAAGTA	27	4-7	27	7-15	34-35	10-11	27-29	25-26
	GGATCAAGTGATTCAAGTACAATTAGGGGATCAAGTGATTCA<br>ATTAGGGGAGCAAGTGATTCAATTAGA	69	4-6	69	3-11	33-34	9-10	26-27	28

**Northern Red Sea**	TAGAATTCGGGGATTAAGTGATTCAAG	27	8-20	27	5-12	34-35	9	24-27	28-30
	AAGTAGGGATTAAGTGATTCAAGTACAATTGGGGGATTAAGTGATTC	48	3.1	47	19	34	7	26	30
	CAATTAGGGGATCAAGTGATTCAAGTACAATGCGGGCATCAAGTGATT	48	6-7	48	16-17	33-34	9-10	27-28	27-28
	AATTGGGGGATTAAGTGATTCAAGTACAACTAGGGGATTAAGTGATTCAAGTAC	48	5-9	54	13-17	34-35	9-10	25-27	28-29
	CAATTAGAGGATCGAGTGATAAGTACAATTAGAGGATCAAGTGATTCAAGTA	51	1.9	52	6	36	10	27	24
	AAGTGATTCAAGTACAATTAGGGGATCAAGTGATTCAATTAGGGGAGCAAGTGATTCAATTAGAGGATC	69	2.6	69	0	33-34	8	29-30	27
	AAGTGATTCAAGTACAATTAGGGGATCAAGTGATTCAATTAGGGGATCAAGTGGTTCAATTAGGGGATC	69	3.5	69	9	34	9	28	27
	CAATTAGGAGATCAAGTGATTCAAGCACAATTCGAGCATTAAGTGATTCAAGTAGAATGAGGGCATCAAGTGATT	75	3.3	75	5	33	9	27	28
	CAATTAGGGGATCAAGTGATTCAAGTACAATTAGGGGATCAAGTGATTCAATTAGGGGATCAAGTGATT	75	5.3	69	6	33	9	27	28
	ATCAAGTGATTCAAGTACAATTTGGGGATTAAGTGATTCAAGTAGAATGAGGGCATCAAGTGATTCAATTAGGAT	75	5.9	75	8	34	10	26	28
**Southern Red Sea**	AATTAGGGGATCAAGTGATTCATC	21	8.9	24	16	33	10	29	26
	AATTCGGGGATTAAGTGATTCAAGTAC	27	8-17	27	7-10	34-35	10	24-27	28
	CAATTAGGGGATCAAGTGATTCAATTAGAGGATCAAGTGATTCAAGTA	48	2.5	48	0	34	10	28	26
	AAGTGATTCAAGTATAATTAGGGGATCAAGTGATTCAATTAGAGGATC	48	4.8	48	12	33	9	29	27
	AAGTGATTCAAGTACAATGCGGGCATCAAGTGATTCAATTAGGAGATC	48	5.8	48	17	34	10	26	27
	TAAGTACAATTAGGGGATCAAGTGATTCAATTAGGGGATCAAGTGA	48	5.6	46	7	34	9	29	26
	AAGTGATTCAAGTACAATTAGGGGATCAAGTGATTCAATTAGGGGATC	48	5.8	48	5	34	9	28	27
	AAGTGATTCAAGTACAATGCGGGCATCAAGTGATTCAATTAGGAATC	48	6.8	47	16	34	10	27	27
	AATTCGGGGATCAAGTGATTCAAGTACAACTAGGGGATTAAGTGATTCAAGTAC	48	7.4-7.9	54	13-14	34	10	27	28
	AATTCGGGGATCAAGTGATTCAAGTACAACTAGGGGATTAAGTGATTCAAGTA	48	7.9	54	12	33	10	27	28
	GGATCAAGTGATTCAAGTACAATTAGGGGATCAAGTGATTCAATTAGGGGATCAAGTGATTCAATTAGA	69	3.9	69	2	33	10	27	28
	CAATTAGGAGATCAAGTGATTCAAGACAATTCGGGGATTAAGTGATTCAAGTACAATTAGGGGATCAAGTGATT	69	5	74	7	34	10	27	27
	CAATTAGGAGATCAAGTGATTCAAGTACAATTAGGGGATTAAGTGATTCAAGTACAATGAGGGCATCAAGTGATT	75	4.7	75	7	34	10	27	27
	AATTAGGGGATCAAGTGATTCAATTAGGGGATCAAGTGATAAGTACAATTAGGGGATCAAGTGATTCAATTAGAGGAGCAAGTGATTATAC	90	2.7	91	10	34	9	29	26
	GGATCAAGTGATTCAAGTACAATTAGGGGATCAAGTGATTCAAGTACAATTAGGGGATCAAGTGATTCAATTGGGGGATCAAGTGGTTCAATTAGG	96	3.7	96	8	34	10	27	28
