# Supplementary material for: Computational Characterization of the mtORF of Pocilloporid Corals: Insights into Protein Structure and Function in Stylophora Lineages from Contrasting Environments
Source: Genes (Basel). 2019 Apr 27;10(5):324. doi: 10.3390/genes10050324 (PMC6562464; doi:10.3390/genes10050324)
Supplement: Supplementary file 1 [file genes-10-00324-s001.zip › Supplementary_26042019/Supplementary_Data_S1_and_S2.pdf]

Supplementary Data S1. Full mitogenomes of *RS_LinA* and *RS_LinB*

>RS_LinA

TGTAAGAAAGACAAAGGGTTAGTCTTTGGGTTCATGCCCCAAATAAGTGAGTTCGAATCTCTCTCTTACACAAAATAAAAATTATAAAAAAAAAAG
AGGATAACTAAGATAAACTAAAATTAGACGATTTTTTTCGAAATGGCGTTCAGTTGCTTATTGGTTGTTTCTCCAGAAAAGTGATTTTTTTAATTTGA
ATTGAAACATCTTATTACTAAGTAAAAATCAAACGAGATTCCGAGAGTGGCGGAGAGTGAAATTGGAGTTGTGCTTGTGGTGGTCATAGATCTTGC
TTAAACATTAGTTTATACTGATAATGAGAGTACTGTAAAGGAAAGTTGAAAGAGAGTTGAAAGGAATTTGAATTTTTTATTTTTGAAGTAGCTTTAA
AAAAGCGTACCTTTTGTATAATGGGTAAAAGAGATTTATTTGGCATATTTAAAATGGAAAATTTTAAGTAATTTTTTTTTTAGTCAAATTTCCCGAAA
CCAAGTGATTTAATCATAAATTGTGTGAGCAAAAACTGGTGATTGTGGCAAAAATCTCAGAAAATTTGTGATTAGGGGTGAAAGGCTAATCGAACT
TGGAAATAGCTGGTTTTCTGCGAAAACTATTTAAGTAGTGTTCTTTTTTTTTTTATTTTAATTGTCATTTTAAAAAAAACAAAAATAAAAAAAAAGTA
AACAAACAATAAATGAAAAGATTTTTTGTTAAAAGAGAAAGCTCAAATCAGAAGCTATTGTCATTTTTTTTTTCTTTAGTTTAAAAAAAAAATTAAAT
TTAGACAATAAAAATGTAGGCTTGGAACCAGCCATCTTTTAAAAAGTACGTAGTTGTTTATTTAAAAATTTTAAATTTTTTTATAATTTTGATGGTTA
AAATAAAACAGAAGCTCTGAAAATAATTATATATTTTGTAGCAGAATAAATTGTTTTCGATATTTGAGTTGATTTCAAAATCTTTTTTAGTTTGAAAA
ATTTTTTTTTAATACATCGTATTCAGTTGTAATAATTTTTACATAAGTAATCTAAATATTATTTTTTTTTTCTTAAGATTACCTTAGTCAAGGATAATAC
AGTTTCTAAAAAAGAAATTTTTTATGAAGATTTTTTATAAAAATATATAATATCGGGAAAAGAAAAAAAAAAGAACTGTTTTTCTAATTAAAGAAA
GAAAAAAAAAAAAGAAAAACATTTTTTTAAGGAACTCGGCAAAGTTAAACTTCGACTGTTTACCAAAAACATAGCTTTTTGATCTTTATAAAAGGTG
AAACCTGCCCGATGGTTGTATCTTAATATGTTTGTCTCGCTTACTAATAAAGACAATTAAATGGCCGCGGTAACACTGACTGTGATAATGTAGCGTA
ATCAATTGTCAATTAATTGTTGACCGGTATGAATGGTACCCCGAAAGTTTTTCTGTCTTAAAAAAATATTCAATGAAATTAAATTTGTAGTGAAGAT
GCTACATTTTAATTGTTAGACGAGAAGTCCCCATGGAGCTTTACTGTAAGTTTATATATATAAATTTTTTTTTTTATAAGCAAGACAGTTTTGTTGGGG
CGACAGTTTTTTAAAAAGTAACGAAAATGAACTATGACGCATGTTTAACTTTGAAATTTTTTTAATTGATGAGACATTTTTGGTGTGTTTTTTGATCCG
TTATTTTGAATGAAAAAAATAACGAAAACAAATAAAAGTTACCCTGGGGATAACAGCGCAATAGTGTTTGAGAGTTAATTAACGACAGTGTTTGCG
ACCTCGATGTTGAATTGTAACGTCCTACGGTGTAGTCACTCGTAAGGGTTGGTTTGTTCATCCATTAAAGTTGTACATGATTTGAGTTAAAAGCGTGG
TGACACAGCTTGGTTTCTATCTACAATTAGAAAACAAAAATATGTTGTTTTTTCGTACGAAAGGATCAAAAATCAATAGTTCCCTTAATATAACTAT
TCTTTAAATTAGGATTGCTTCTAAAAATAAAAAGAAATATTTTTGTTTGTTGTTTTTATTAATGTATCTTTTAGTTTTATTTTTTCCTTTAATTGGAGCTG
TTTTAACAGGTTGTTTTGGAAGAAAAATTGGAGAAAGAGGAGCGGGGATTTTAACTTCAAGTTGTTTAGTTTTTAGTTTGTCTTATTCTTTTTTAATCG
CGATTGAAGTTTTATTTAATTCAACAACAACGTATTTGAAATTATGAAAATGATTTGATTCGGGATTGTTTGTTGTTTTTTTTGGTTTTCAATTTGATGA
TTTAGTAGTTATTATGTTATTTGTTGTTTTTATTGTTTCTACTTTGGTTCATATTTTTTCTATTGCTTATATGCGGGGGGATCCTCATATTCCTCGATTTAT
GACATATCTTTCTTTGTTTACTTTTTTAATGGTTTTGTTGGTAACTAGTGATAATTTTCTTCAATTATTTATTGGGTGAGAAGGGGTTGGCCTTTGTTCTT
ATTTATTAATTAATTTTTGATTAACTCGATTAGAGGCAAATAGAGCTGCTATTAAAGCAATGTTAGTGAATAGAATTGGTGATATTGGGTTGCTTTTA
GCAATGTTTTTACTTTGGGATCTTTTTGGATCTCTAGATTTTTCTACAATTTTTAATTCTATTTTTTTTTCTAATCAAATATTTTTTATTTGTTTATTTTTAT
TTTTTGGGGTTATGGGAAAATCTGCTCAATTAGGATTACATACTTGATTACCGGATGCAATGGAAGGTTATTGGGCCTTTTAATTAAAAAATTAATTA
AAAAAATTACTATACATTAAAAAAACAATTTATTAGTAAAATAATTGTTTATTAATGGACAATAAAATATTTATTTTAATGTCTAAGAGACTTTATG
TGATTTACTTTTTTTTATTTGATTAAATCTATTATTATTATTATTCCTTTACTTATTGCTGTTGCATTTTTAACTTTAGCAGAACGAAAAATTTTAGGGTA
TATGCAAATAAGAAAAGGACCAAATGTTGTTGGAGGGGGGCTTCTTCAACCTTTTGCAGATGGGGTGAAATTATTTATTAAAGAAATGATTCTTCCC
CATCAAGCAAATAAATTTGTTTATCTTTTGGCTCCAGTTATATCTTTTACATTAGCTTTCTTTGTTTGAGGTTTTCTTCCTTATGAGAAAGGAGTGAGTA
TTAGTGATTTTAAAATTAGTCTTTTATGAATTTTGGCTATTTCTTCTATTAGTGTTTATGCAATTTTAATGTCTGGATGAGGAAGCAAGTCTAAATATG
CTTTTTTGGGTGCTATTCGGGCGGCGGCTCAAATGATTAGTTATGAGGTTTCAATTGGGCTAATTCTTATTTCGGTTTTATTATGTGTTGGTTCTTTAAG
TATTACTGAAATTGTTTTAGCGCAAAATAATGGTATTTGGTTTTTTTTTCCACTATTTCCTGTTACAATAATGTTTTTTGCTTCGATTTTAGCAGAGACT
AATCGTGCTCCTTTTGATTTAACAGAAGGAGAGTCAGAGCTTGTTTCGGGATATAATGTTGAGTATGCTTCGATGTCTTTTGCTTTATTTTTTCTTGCT
GAATATGCACATATTATTTTAATGAGTTGTTTAACAACGATTTTTTTTTTGGGAGGATGGCTTTCTCCGATTCCAGATTTTAAGGGTGGGGCTGGATG
GTTTGGTTTTAAAGTTGTTTTTATTATTTTTTTTTTTTATTTGAGTAAGGGCTTCATTTCCTCGAATTCGATATGATCAGCTTATGGCTTTGTTATGAAAAG
GGTATTTGCCTTTAAGTTTAGGAATGGTCATTTTTGTGGCTAGTGTTCTCTTTGGGTTTAATGGCCCCCCTCCTATTTAAAATATTTTAATTAATTATTA
TGAAAAAAAAAAAAGAACTAACAGATTTGCAAGTGCCATTACGAAAAAAAGATCCTATTTTATTTTTAATCAACGGATTTTTAGTTGATTTGGTTTC
TCCTTCTAACATTACTTATTTATGAAATTTTGGATCTTTATTGGGATTGTGTTTGATTTTACAGATAGTCACTGGGTGTTTTTTGTCTATGCATTATTGTT
CTGATGTTAATTTTGCTTTTGCTTCAATTGGTCATATCATGCGAGATGTTAATTATGGGTTTTTATTAAGATATCTTCATGCTAACGGTGCTTCTTTGTT
TTTTTTTTGTCTTTATGTTCATATTGGTCGAAGTTTATATTATGGGGGGTATTTAAAATTTCATGTTTGAAGCATTGGAGTTGTTATTTTTTTATTAACAA
TGGCAATTGCCTTTATGGGCTATGTTTTGCCTTGGGGACAAATGTCTTTTTGAGGGGCAACTGTAATAACTAATTTATTGTCTGCTATTCCTTATTTTG
GGATTGATATTGTTCAGTGAGTGTGAGGCGGGTTTAGTGTTTCAAATGCAACATTAAATCGGTTTTTTAGTTTACATTTTTTACTTCCTTTTATTTTAGT
TTTTCTTGTTATTCTTCATTTAGTTTATTTACATGTTGATGGGTCTAATAATTCAACAGGATTAAATTCTTCAATTGACGGAGTTTCATTCCATACTTTTT
ATACATCAAAAGATTTTTTTGGTTTTTTTTTTCTTTTTTTTTTATTTGTTTTTTTGTTTTTTTTTTGCCAAATTTATTAGGAGATGCTGAAAATTTTATTCA
AGCGAATTCTTTGGTTACTCCGGTTCATATTCAACCAGAATGATATTTTTTATTTGCTTATGCAATCTTACGTTCTATACCAAATAAATTAGGTGGGGT
TATTGCAATGTTTTGTAGTATTCTTATTTTATTTTTTTTTATCTATTTTACATCAAAGTCTTTTAAAGGGGCTTTTTTTTCGTCCTTTAGGTCGAATTGCATT
TTGGTTTTTAATTATTGATTTTGCTTTATTAACTTGAATTGGATCACAAGTTGTAGAAGAACCTTTTATTTTAATTGGTCAAATACTCTCTTTTTTTTATT
TTTTTTATTTTTTAGTTTTAATACCAGTTTTGGGTATTATTGAAAATCAATTATTAAATAAAAAATTAATGAAATTTTTGTTTTAGGGGGTTTTCTTTTGA
GCTTCGTGGTTTTAAGTCTTCGTTATTTTCTTTTAAAACTTTCTCTTTTTTATTTATTATTATTTTTTTGTTTTTTTTTTTTTAAAATTAGAATGGGCAAAGCT
TTTGGTTTTAATTGGTTCTTTTGCTATCTTTCTTTTATTTGGACCAAAAAAAGAAGAACAAACAGATGTGCCTATTTTAAGTTTAATTATTGTTTTTGGA
GTTTTTTGTTTAATTTCTTCAAGTAATTGACTTTCTATTTATTTAACCATTGAACTTTTTACTCTTTGTTTTTTTATTTTGATTGCTCGAGGGTCTGGTTAT
AGTGTGGAAGCAGGATTAAAATATTTTATTTTGGGTGCTCTTTCTTCTGGTTTATTTTTATTTGGGTGTGCTTTATTATGTGGTATTGGGGCTAATATAC
ACTTTTCTCATATAGAACTTCTTTTTAATTCAAAACAAGTTTTTTCTGCTGTTTCGATACCAATTGGGTATCTTTTAATTATTGTTGCTCTTTTTTTTTAAA
TTATCAGTTGCTCCTTTTCATATGTGAGTTCCTGATGTTTATGAGGGGGCACCTACTAAAATTGTTCTATTATTGGCTATTGTTCCAAAAATAGGGTTT
TTTTCTCTTATAATTTCAATTGGATTGCCTGTAAATTTTTTTTTTTTAGGGATTCTTTTTTCTTTGTTTGTTGGAGCTTTGGGTGCTTTAAATCAAACTAA
AATAAAACGACTTTTGGCTTATAGTGGGGTTGGTCATATGGGCTTTATTTTATGAGGTTTGGAAAATGGTTCTTTTGAAAGTTTACAAGCTAGTCTTG
TTTATCTTTTTATATATATTGTGATGACAATTTGTGCTTTTTCTATTATATTAAGTTTTAATGTTTATAAAAATTTGCTTGTTGAATTTAGTGGACTTTCT
CGATACTTACCTTTTTTTTCGATTACCTTAGGTGTTCTTTTTTTTTCTATTGCAGGAATTCCTCCTTTTGCTGGATTTTTTGGAAAATGATTTATTTTGTTA
TCTGGAATTCTTTCTAAATCATATTTTATTTTTTTTTTTGCTGTTTTTTGTTCTGTTATAGCTGGGGTTTATTATATTCGAATTATAAAAATACTTTTTTTT

CAAAAAAATTCTTTTCTTTTAATTACAATTAAGGCTTTAAAAAAAGAATCTAAATTAAATTTTAAAAAAGTTTTTTAATTGGTTTTTGTTTTTATTTTA
TTCTTTTTTTTTTTCTTTCTCCACATTTTCTTTTTTTTTTTTTTTCTCAAATAATTTTTGATTTATTTTAAAATGGAATTTTTTTCTTTTTTTTTTATTTTAGGG
ATAATTGGCTCAGGAGTAATGGTTGTTTCAGCGTTAAATCCTGTTCTTTCTATTTTTTGATTGGTTCTTGTTTTTATAAATTCTGCAGTTTTTTTCCTTTT
ACTAGGAATAGATTTTCTTGCTTTGATGTTTTTACTTATTTATGTTGGGGCAATAGCTATTTTATTTTTATTTGTTATTATGTTATTAAATCTAACTGACT
ATCCTCCGGTTTTAAAAAGAGAGGTTGATATGACAAATTATATACCAATTGGATTTATAATTGGGATTTTTTTTTTTTCAGAAATTGCTTCAAGTGGA
TTATTTTTGGGTTCTTTTCAAATAGAGAATTGAGACCTTTCTTTTCCTTGATTTCTTATTTCTTATCATAATATTGAGGCCTTAGGGCAGATTTTATATGT
TTCTTGTTTTTGTTTATTTCTTTTGGCCAGTTTTATTTTATTAGTTGCTATGATTGGTGTGATTGTATTAACTCAAGAAACAGAATTTTTAAGTAAAAAA
CAAGATCTTTTTTTTCAAATAAATAGATAATGAATAGCTCTTCTTATTTTGAACAATTTAACATAGTGTGAATGTTTGGTTTTACGAATTCAACAATA
ATGATGACTTTTGTAATTATTGTTGTTTTATTACTTTTTAAAGGAATTGAATTAATTCCAAAAAGATGGCAATCGGTTTATGAATATCTAGAAAATTAT
TTTTATTATATAACAGTGCAAAATTTAGGAAATGTGGGTTTAATATATTTTTCTTTTATTGTTTCACTTTTTGTTTTTTTAGTTTTTTTAAATATTTTAGGC
TTATGTCCTTATGTCTTCACACCGACAACCCATATTGTTAATACGCTTGGATTTTCTTTTTCTATTGTTATTGGAGTTACTTTTTCTGGATTTTATAAATT
TAAAAAAGACTTCTTTAGCATTTTAATGCCTAGCGGCGCTCCTTTAATTTTAGCACCACTTTTAATTTTAATAGAAACAGTAAGTTATATTTCTCGAG
CTGTTTCTTTGGGCATTCGTTTAGCAGCAAATCTTTCTGCCGGGCATCTTTTATTTGCAATTTTGGCAGGATTTGGTTTAGTTTTTAAATCAGCAATGTC
TATAATGGTGTTTATTACTTTATTGGAGGTGGCCGTTGCGGTGATCCAAGCGTATGTGTTTTGTCTGCTGACATTAATTTATTTAGCAGACACAATTTT
TCTACACTAATCATGAGCGGGGTAGAAATTTTTTTATGCTCATTAATGGGTGTGTCGGTGTGTTGTAATTATGCGTTCATGATGGCTTGATACAGCCA
ATCTTCTCAAAGGTGACTTCAGCATCAAATGGATCTTCCAGAGAATACTGAATACGTGCATCCTTGAACTTATTATAGTTATGTGCGCCGTCATGGT
ATTGTTCATAATGGTGGGGTACAAGTAGATCAAGAATCCAATGGTGCTGATCGGCCAAGAGATGTAATTTTTATCGATTTAGAAAGGCGACAAGTG
CCCTTAAGGGGTCGGCGAGTTTTTAGGGTGGCGGAAAATGACGAGAGTTTGTGTGATAATGGTAATCGCTTGTGTCATGACGAGGGTTTGTGTGATA
ATGATGATCGTTTGTGTGTTCATGGCGAGAGTTTGTGTGATGATGATAGTTTGTGTGATAATGACGAGTTTCAAGATGGGGTTTTGCATTTAAGTAAA
AGTGCAAGTGAGTCAGAAAGTAGAGGTAGTTTGAGGGAAAGTGTAATTAAGGCATTAAGTGAGTCAGAAAGTAGAGGTAGTTTGAGGGAAAGTAT
AATTAAGGGATTAAGTGATTCAAGAAGCGAAGACGAAGCGTTAAGGGCAGTAGAGGTAGAAACACATTTAAGTGGTGGCCAGTTAGCGCGTACA
AATCAAGGTGAGGCAGTTGAATCGACTCTTAGTTCGATCCCTTTTGAGGTTGAAGGGATGATGGTTCCTGTGTGAAATCAATTTGGCATGCTGTTTA
ACATATTGGGAGATAAGCCCCTGGTGAGCTGCATTTTTTTATGTTATCAGCTTTTAAAAATAACAAATCAAATGCCAAACCCATGGGTTTATAGGTG
ATTTATTTTGGTATATATTTTATTAATCTTTGTTTTTTTAATGATTTAGTTATCTTTTATTTAATAGAGTTGAGAGAAAAAGTTTTAAAATGGTTCTTATT
TATTTAATTGTTATTATGGCATTAGTTAACATCATAGGGACAGCGAGGGAGAGAAGATGTGTTTTAAAAAAGCGGGCTTTAGAGTGGTCTTTGGCTT
TATTGTTTAGTACTTTAGTTTTTTGGGGTGGATTTGACGGAGAAAGTCATTTTCAATTTTTTAGTTTAGTAGAATGAAATATATTTTCTACTTTAGATTG
AGGCCCGATTGTTTTTGCAGTCGATGGTGTTTCTTTGGTTTTTTTATTTTTAACGACTTTTTTAATTCCAATTTGTATTTTAATTAGCCAAAAATCAATA
AAATTTTTATTTAAAGAATTTCTTTTGTGCTTGTTTTTTTTAGAAGTTTTATTAATAGGTGTTTTTATAGTGTTTGATCTTCTTTTGTTTTATCTTTTTTTTG
AGGGGATATTAATACCAATGTTTTTTTTAATTGGTATTTGAGGCTCTCGAGAAGAAAAGGTTCGTGCTTCTTTTTATTTTTTTTTTTTTACTTTTATAGG
CTCTCTCTTTTTTTTTTTTATAATACTTTTTTTATATCAAAGGATTGGAACAACAGACTATTTTCTTTTACTTAATATTAAATTATTTTTAAATATTCAAA
AATGAGCCTTAGTTGGGATTTTTCTTAGTTTTGCGGTGAAACTACCTCTTATTCCATTTCATATTTGATTGCCACAAGCACATGTTGAGGCTCCTGTTG
CGGGCTCTGTTATTTTGGCTGGAATTTTATTAAAATTAGGGGGTTATGGCCTTTTGCGTTTTTCTTGACCTCTTTTTCCGGGGGCTTCTTTATATTGGTCT
CCAGTTATTGTTTTTTTTAGTGTTGTTGCTGTTGTTTATGGAGGCTTAATGACATGTCGTCAAATTGATTTTAAACGGCTTGTTGCTTACTCTTCTGTTGC
TCATATGGGACTTGTGCCTTTGGGTCTTTTTACACATGTTATAGAGGGGTTAATTGGGGCTCTTTTTTTAATGTTGGCGCACGGATTTGTTAGCTCTGC
TCTTTTTATTGGAGTAACTTTTTTGTATGATCGCCACCATACTCGTTTAATAAAATATTATCGGGGTTTGACTTTGACAATGCCTCTTTTTGTTATTACA
ATGTTAATTTTGTCTTTGGCGAACATGGGTTTTCCTCTTAGTTGTAATTTTGTTGGAGAATTTTTTTCTTTATTAGCAGTCTTTCAATATCATTATGGAGT
TGGAATGTTTGTTATTTTAGGGGTTCTTTTTTCTGCAATTTATTCTCTTAGTCTTTTTAATCGTATTTCTTTTGGTGGAGGATCTAATTATTTACTTTTTAA
TAGAGACTTAAGTCGACGAGAGATTTTTGTAATTTTTCCTTTTCTTTTAATTATCTTAGGGGGGGGGATTGTGCCTTTTCCTATTATTGATTTAATTAAA
AATAGTCTTGTTTTTAGCCCAGGGGGTTAGTCCTGGGGTTTTAGGGAAAAAAAAATATTTAGTCTAGGTTATACATGCTAGTGTATGCGAACAGGTG
AGGATAAAATAAAAAAATTTTTTTTATTTGTTGTATTAAAAAAAGAAAAGTTTTTTATTCTTTTTTTGAAGATGCATAGTTTAACCACATTTTCACTGA
AACAAAAGAAAACCTTTGGCAGCAGTAATGGATTTTATGTAATATACGAAAGTAAGACATAGGTGGGATAAAAAAAATCTGTCAAATATAAGTGC
CAGCAGACGCGGTAAAACTTAAGGATTTATTTTTTAATAAAAAGCAAAAAGCGTGTTAAGGATTTTTAAAAAAAAAAAAAATAAATAGAATTTTTTT
CGTAATTGTGAAATGTTGGAATGAAAAAAAGAATTTTTTATATGAAAATAATTTATTTTTTTTTTCTTAAATACGAAGGTTTTGGGAGCAAACAGGAT
TAGAGACCCTGGTAGTCTATACAGTAAAAGAATATCGCTTGAGTAGTACGGTCGCAAGATTGAAATTCAAAAGACTTGGCTGTTCGGTTGTTAATTA
GAGGAGCGCGTCGCTTAATTCGATAATCCGCGAGAGACCTTACCGAAGTTTGAATTTATTAACAGGTGTTGCATGGCCGTCGTCAATTTCTGTTTGA
GACAAAAAGGGGTTTAATCCTATCAAGGTTGAAGTCAGGTATTATGACCCTTATATTTCGGGGTTAGGCGCGCTACATTTTTCTCTTAGAAAAGGAG
GGGTTCGGATTGTTTTTAAAATAAGATAATGAAGTTGGATTTAATAGTAATTGAAAAGTAGTGCGTTTCAATGAACGTGAAAACAATGTTAGTACA
AATAGCCCGTCGCCAGCCCAGAAATAGGCTGTAAGTCGTAACATAGTAAGAGTGAGGGAACTGGCTCTTGATGCAGTTTCATTCGTATCATTTAGTT
GAGCCTTCTCCATGGCCTTTTGTTGGAGCAATTGGCTCTTTTTTTATTACTGTTGGTGCAGTGGTTTTTTTTCATTATGGGTTTAGTTTTTTTTTATATTTG
GGGCTTTTGGTTGTTGTTGGAGTTATGTTTGTTTGATGACAAGATGTTATACGAGAATCAACTTTTCAAGGTCACCATTCTTTAATTGTAAAACAAGG
GATTAAATATGGTATGATTTTATTTATTCTTTCAGAAATTTTGTTTTTTTTTTCTTTTTTTTGGGCTTTTTTTCATAGTAGTTTAGCACCGGTTGTTGAACT
TGGAGTTGTTTGGCCTCCACAAGGAATTGTTGCATTAAATCCCTTTTCTGTTCCTTTATTAAACACTGCTGTATTATTAAGTTCTGGGGCAACGGTTAC
ATGGGCGCACCATGCAATACTCTGTGGTTTAAAAAAAGAGGCTCAACTTGCTTTATTTTTAACTCTTTTTTTGGGTGTTATGTTTACGGGGTTGCAAG
CATTTGAATATTATGAGGCGCCTTTTACTTTATCTGACTCTGTTTATGGGGCCACTTTTTTTGTTGCGACAGGGTTTCATGGACTACATGTTATAATTG
GTACTACTTTTCTTTTTATTTGTTTTTTACGTTTACTCTCTAATCAATTTACTCGTCGTCAACATGTTGGGTTTGAGGCCGCGAGTTGATACTGGCACTT
TGTCGATGTGGTTTGATTATTTTTATATTTATGTATTTATTGATGAGGCTCATAAAAACTTCTTTAATGCAAAATCTTTTTGTTTTTACTTTTTTTAGATA
TTTTATAAATGTTTTGGCTTGGTTGTCTGTTCTCTGGTTTAATCATCTTTATTATTCGGACAGTCCAGAGACTTGACTTTTAGAGTTTCAAGATGTTGGG
GATCCTATTGTAGAAGAAATTGTTTTTTTTCATGACCAAGTTATGTTTTTATTAATTATTATAGTTACTGTTGTTTTATGACTTTTTGTTGAATCTTTTAA
AAATAAGTTTTATGATCGTCATTTAATCGATGGTACATTTTTAGAAATTGTTTGAACGATTATTCCTGCTGTTATATTGATTTTTATTGCATTGCCCTCT
CTTAAATTATTATATTTAATGGATGAGGTTATTTCTCCAGCTTTGACAATTAAAGTTATTGGGCATCAATGATATTGATCCTATGAATATTCTGATTAT
GAAGGGGATACGTTAGGGTTTGATTCTTATATGATTCCTACTTCGGATTTAGTTTCTGGAGAGAATCGTTTGTTAGAAGTTGATTATAAACTTTTAATT
CCTATTCAAACACATATAAGATTTTTAGTTACTGGGGCAGATGTTTTACATTCTTTTGCAGTGCCTTCTTTGGGGTTAAAAATTGATGCGGTTCCGGGT
CGTCTAAATCAAACTGGTGTTTTTATAAAACGAGCCGGGGTTTTTTTTGGACAATGTTCTGAAATTTGTGGGGCAAATCATTCTTTTATGCCAATTGT
GATAAAGGGAGTTGGTTTAAATGAATACGTTCAATATTTAAATTATTTAAAATGTATTATAAATACTTAGTTATTATTATTATTTTATTTTTATTGGGG
AGTTGGGGTATAATTTTAAATAGAGGACATTTTATTATTATGCTTGTTTCTATTGAATTAGTTTTATTATCAACTTTTTTTTTTTTTTTAATAAGCTCTAA
AGAAATAGATCTTTTAATAGAACAAATTTTTATGATTATGGGATTAACTATTGCTGCGGCAGAATCTTCAATTGGTTTAGCTATTTTAGTTGCATATT

ATAGAATTCGAGGAACAATTGTTTTAAAATCTTTTAGTTCTTTGCGAGGATAATGATTAATCTTTTTATTTTTTTTTTTCTTATTTTTATTTTGGCGGGGT
TATTTATATTTTTTTCTTTTTTGATAGGGGAAAAAAATTCCAGATCGAGAAAAGGTTTCTGCTTATGAATGTGGTTTTGCTCCCTTTAATTTTTTAGGGCA
CCCTTTTTCAATACGTTTTTTTTTAATTGGTATTTTATTCTTGATTTTTGATTTAGAAATCTCTTTTTTTTTTCCCTGATGTGTTTTATATAATTCGACTGCT
CCGTTTGGGTTTTGAACTATGATAGGTTTTTTTTTTGTATTAGTTTTGGGTTTGATATACGAATGAGTAATGGGGGGGTTAGAATGGGAGTAAAAAGA
GAGTAAAAGAAAAAGTCCTATCTATTATGAAAATAATAGAAATTTTATACCAACTCCGGTTTCTGCTTTAATTCATGCTGCAACGATGGTTACTGCA
GGTGTTTTTTTATTAATTCGTGCTTCTCCTTTATTTGATGTTGTTCCTCTTATATTAATTATTATTTCAATCGTTGGGGTTTTAACCGTGTTTATTGCAGG
GACAATTGGTTTAGTTCAAAATGATTTAAAAAAAATAATCGCTTATTCTACTTGTAGTCAATTAGGTTATATGGTTGTTGCTTGTGGGCTTTCTCATTA
TGCAATTAGTCTTTTTCATCTTATGAATCATGCTTTTTTCAAAGCTTTATTATTTTTGAGTGCAGGGTCTCTTATTCATGCGGTAATTGATGAACAAGA
CATAAGAAAAATGGGGGGTTTATTATCTTTTCTTCCTTTGACTTATGTTTTTTTTTTGATAGGCTCTTTTTCTTTAATGGGGTTTCCTTTTTTAACAGGAT
TTTATTCAAAAGATTTAATTTTAGAATTTGCTTTTGGACAATTTTATTTAATTTTTGTGTATTGATTAGGTTGTTTTTCTGTTTTATTAACAATTATATAC
TCTATTCGTTTAATTTATTTAGTTTTTTTATCCAATATTAATTTAAAACGAGCAAACATTTTTTTTCTTAAAGAAGGAGAATTTTTATTTTTAATTCCTTT
GGGTATATTAACTTTAGGAAGTGTTTTTTGAGGCTATTTAAGTAAAGAAATAATTTGGTCTTTTCAAATAGATGTTTTTTCAATACTTTCTTTAAAAAT
AAAAATATTTCCAATTTTATTTTGTTTTATTGGACTTTTTGGAACGATATTTTTTTTTTTTTTTTTTTCTTCTCAAATTTTTGGCTATCCCCTTCAGGTGAT
CAGATCTTCTGTTTTTTCTCTTTATAATTTTTTTGGTTCTGCTTGACAAATAAATTTTTTTTTTAATTTTTTCTTTATAAAAAAAATATATAAAATAGGAC
ATCTAATTACTAATTTAACAATTGATAAAGGTTTATTAGAGGTTGTTGGGCCTAGGGGTGTTGTTCAATTTTTTATTTTTCAAACTCAAAAGTTGAGTA
GTTTACAATCGGGGTTAGTATTTAATTATGCTTTAGTTTTTTTTCTTGGGATACTTTTTTTAATTTTTGCACTTTAATTTAAATTTTTGAAGGGGTTAAGT
TAAAGTAAACTGTTGATCTTCAAAATCAGCTATGTAGGTGCAAGTCCTACACTCTTTGAGTGCCTCAGTTGAAAGTCGGCTTTTATAAGGTACAATA
TTGATGGTGTTTTTCTGTTTTGTTTTTATTGTTAATTTTTTTTTGAGGTTGTTATTTTTCCTTTAATAAAAAATAATTGATGTATAAGAAAGTTCTTAATGA
AGTGCAATGGCGCCATCTTAACAAAAAGTTGGTTACAAAAAGAACTTTATAAAAAAAAAAAAAAAAAGTATGGTGTAATACTAAACTTTTTTAAAAA
ATCCCTAAATTATTAAATGATGAGTTATATTAAATATTTATGAAAAAAAATGGGCTAGTATTGGGTTTATTAGTAAGTTGCAAGTTTTTTTGATGAGGT
ATAGATTGAAAATTATTTTGATTATCGGTGGTGTAATCCTTCTCGTTATAGGAATACTCCTAGCTGTCCTAGGAGTGGATTTCGAGGGGAGTTTTTAT
AAAAGAATTAAAAAAAAAATTGTTTGTTGACGCTGCGGGTCCGCCCCGTAAACGTGCTAGGGTTGAAGAAGAACGATCGTCAAATATGCCCGGGAA
TATTGAAAATGAAATCGTGGGCGGAGCCGAAAACGAAGAGCTACCGGCGGCGTTTGCACCTTTAGCCAACCCAGTGAGTACCGGAGTTCGAGAAA
CCGAGGGAGATTTGGGTTATTCAGATGAGGTTTGGGATGCATTAAATGGATTTGTATTTCAATACTCTACAGAAGATCAAGAAGACGAATTAGTAG
AGATGCTAATTGGGTGAGCAAACAGTGTAACGGCCGGAGAGTCAAACGAGGATATAGTAGATGTGCTAGGGGAGTGGGCAGAGGGTAACATCCC
CGAAGATTCAGGCGACGAATCAGAGGAGAACGCGTCTGAAGATTCAAACGACGAATAGGCGGAGGCCCCCATTTATTCAATATGTAAGAGATCG
GGGGGGGGTTGTGGGACCTCGCCCCGGGTATTTGAACGATATGGTCTCTAATTATTGAATTCGATGGGGGTTTTCAACCAACCATAAAGATATTGGT
AGTTTGTATTTAATTTTTGGTGGAGGTGCTGGTTTAATTGGAACGGCGTTTAGTATGCTTATACGACTAGAGCTTTCTGCGCCCGGAGCAATGTTAGG
AGATGATCATCTTTATAATGTAATTGTTACAGCGCATGCTTTTATTATGATTTTTTTTTTTGGTTATGCCGGTTATGATTGGGGGGTTTGGTAATTGATTG
GTCCCATTATATATTGGGGCGCCGGATATGGCGTTTCCCCGACTAAACAATATTAGTTTTTGACTTTTGCCCCCTGCGCTTTTTTTATTATTAGGCTCT
GCTTTTGTTGAACAAGGGGCGGGGACGGGGTGAACAGTTTATCCTCCTCTTGCTAGTATTCAAGCACACTCCGGATGTTCGGTTGATATGGTTATTTT
TAGTCTTCATTTAGCTGGGGTTTCTTCTATTTTAGGTGCTATAAACTTTATTACTACAATTTTTAATATGCGAGCCCCGGGTGTGTCTTTTAATAAACTA
CCTTTATTTGTTTGATCTATTTTAATAACAGCTTTTTTATTACTTTTATCTTTACCTGTTTTAGCTGGTGCTATTACTATGTTGTTAACAGATAGAAACTT
TAATACGACTTTTTTCGATCCAGCGGGTGGCGGGGACCCAATATTATTTCAGCATCTATTTTGATTCTTTGGGCATCCAGAAGTTTATATTTTAATTTT
GCCTGGTTTTGGTATGATTTCTCAAATAATCCCGACTTTTGTTGCTAAAAAACAAGTTTTCGGGTACTTAGGAATGGTTTATGCCATGCTTTCTATTGG
GCTTCTGGGATTTATTGTTTGAGCTCATCATATGTTTACTGTTGGGATGGATGTAGATACAAGAGCATATTTTACTGCTGCTACTATGATTATTGCTGT
GCCAACTGGGATTAAAGTTTTTAGTTGGTTGGCAACTATTTATGGAGGTGTTCTTAGGTTAGAGACTCCAATGCTTTGAGCTATGGGGTTTGTTTTTTT
ATTTACAGTTGGTGGTTTAACTGGGGTTGTATTAGCAAATAGTTCTCTTGATATTGTTCTACATGATACATATTATGTAGTTGCGCATTTTCATTATGTT
CTTTCTATGGGGGCTGTTTTTGCTATCTTTGGGGGATTTTACTATTGAATTGGAAAAATAAGTGGTTATTGTTATAATGAATTTTTTGGGAAAGTTCAT
TTTTGATTAATGTTTATCGGGGTTAATTTAACTTTTTTCCCTCAACATTTTTTAGGTTTAGCAGGATTTCCAAGACGATACTCGGATTATGCAGATGCT
TTTTTGGGTTGAAATTTAATAAGTTCTTTAGGGTCTATTATTTCTATTTTGAGTGTTGTTTGGTTTTTATATATTGTTTTTGATCTTTTTGTTACAGAAGA
AAAATTTTTGGGTTGAAAAGAAGGATTTTCTTTAGAATGAATTCATTCTTCTCCCCCCTTATTTCATACTTATGAGGAGTTGCCTTTTGTACAAAAATA
AATAATTTTTAGAATAGTGCCGGGTTTATATACCGGTTT

>*RS_LinB*

TGTAAGAAAGACAAAGGGTTAGTCTTTGGGTTCATGCCCCAAATAAGTGAGTTCGAATCTCTCTCTTACACAAAATAAAAATTATAAAAAAAAAAG
AGGATAACTAAGATAAACTAAAATTAGACGATTTTTTTCGAAATGGCGTTCAGTTGCTTATTGGTTGTTTCTCCAGAAAAGTGATTTTTTTAATTTGA
ATTGAAACATCTTATTACTAAGTAAAAATCAAACGAGATTCCGAGAGTGGCGGAGAGTGAAATTGGAGTTGTGCTTGTGGTGGTCATAGATCTTGC
TTAAACATTAGTTTATACTGATAATGAGAGTACTGTAAAGGAAAGTTGAAAGAGAGTTGAAAGGAATTTGAATTTTTTATTTTTGAAGTAGCTTTAA
AAAAGCGTACCTTTTGTATAATGGGTAAAAGAGATTTATTTGGCATATTTAAAATGGAAAATTTTAAGTAATTTTTTTTTTAGTCAAATTTCCCGAAA
CCAAGTGATTTAATCATAAATTGTGTGAGCAAAAACTGGTGATTGTGGCAAAAATCTCAGAAAATTTGTGATTAGGGGTGAAAGGCTAATCGAACT
TGGAAATAGCTGGTTTTCTGCGAAAACTATTTAAGTAGTGTTCTTTTTTTTTTTTATTTTAATTGTCATTTTAAAAAAAACAAAAATAAAAAAAAAGTA
AACAAACAATAAATGAAAAGATTTTTTGTTAAAAGAGAAAGCTCAAATCAGAAGCTATTGTCATTTTTTTTTTCTTTAGTTTAAAAAAAAATTAAAT
TTAGACAATAAAAATGTAGGCTTGGAACCAGCCATCTTTTAAAAAGTACGTAGTTGTTTATTTAAAAATTTTAAATTTTTTTATAATTTTGATGGTTA
AAATAAAACAGAAGCTCTGAAAATAATTATATATTTTGTAGCAGAATAAATTGTTTTCGATATTTGAGTTGATTTCAAAATCTTTTTTAGTTTGAAAA
ATTTTTTTTTAATACATCGTATTCAGTTGTAATAATTTTTACATAAGTAATCTAAATATTATTTTTTTTTTCTTAAGATTACCTTAGTCAAGGATAATAC
AGTTTCTAAAAAAGAAATTTTTTATGAAGATTTTTTATAAAAATATATAATATCGGGAAAAGAAAAAAAAAAAGAACTGTTTTTCTAATTAAAGAAA
GAAAAAAAAAAAGAAAAACATTTTTTTAAGGAACTCGGCAAAGTTAAACTTCGACTGTTTACCAAAAACATAGCTTTTTGATCTTTATAAAAGGTG
AAACCTGCCCGATGGTTGTATCTTAATATGTTTGTCTCGCTTACTAATAAAGACAATTAAATGGCCGCGGTAACACTGACTGTGATAATGTAGCGTA
ATCAATTGTCAATTAATTGTTGACCGGTATGAATGGTACCCCGAAAGTTTTTCTGTCTTAAAAAAATATTCAATGAAATTAAATTTGTAGTGAAGAT
GCTACATTTTAATTGTTAGACGAGAAGTCCCCATGGAGCTTTACTGTAAGTTTATATATATAAATTTTTTTTTTTATAAGCAAGACAGTTTTGTTGGGG
CGACAGTTTTTTAAAAAGTAACGAAAATGAACTATGACGCATGTTTAACTTTGAAATTTTTTTAATTGATGAGACATTTTTGGTGTGTTTTTTGATCCG
TTATTTTGAATGAAAAAAATAACGAAAACAAATAAAAGTTACCCTGGGGATAACAGCGCAATAGTGTTTGAGAGTTAATTAACGACAGTGTTTGCG
ACCTCGATGTTGAATTGTAACGTCCTACGGTGTAGTCACTCGTAAGGGTTGGTTTGTTCATCCATTAAAGTTGTACATGATTTGAGTTAAAAGCGTGG
TGACACAGCTTGGTTTCTATCTACAATTAGAAAACAAAAATATGTTGTTTTTTCGTACGAAAGGATCAAAAATCAATAGTTCCCTTAATATAACTAT

TCTTTAAATTAGGATTGCTTCTAAAAATAAAAAGAAATATTTTTGTTTGTTGTTTTTATTAATGTATCTTTTAGTTTTATTTTTTCCTTTAATTGGAGCTG
TTTTAACAGGTTGTTTTGGAAGAAAAATTGGAGAAAGAGGAGCGGGGATTTTAACTTCAAGTTGTTTAGTTTTTAGTTTGTCTTATTCTTTTTTAATCG
CGATTGAAGTTTTATTTAATTCAACAACAACGTATTTGAAATTATGAAAATGATTTGATTCGGGATTGTTTGTTGTTTTTTTTGGTTTTCAATTTGATGA
TTTAGTAGTTATTATGTTATTTGTTGTTTTTATTGTTTCTACTTTGGTTCATATTTTTTCTATTGCTTATATGCGGGGGGATCCTCATATTCCTCGATTTAT
GACATATCTTTCTTTGTTTACTTTTTTAATGGTTTTGTTGGTAACTAGTGATAATTTTCTTCAATTATTTATTGGGTGAGAAGGGGTTGGCCTTTGTTCTT
ATTTATTAATTAATTTTTGATTAACTCGATTAGAGGCAAATAGAGCTGCTATTAAAGCAATGTTAGTGAATAGAATTGGTGATATTGGGTTGCTTTTA
GCAATGTTTTTACTTTGGGATCTTTTTGGATCTCTAGATTTTTCTACAATTTTTAATTCTATTTTTTTTTCTAATCAAATATTTTTTATTTGTTTATTTTTAT
TTTTTGGGGTTATGGGAAAATCTGCTCAATTAGGATTACATACTTGATTACCGGATGCAATGGAAGGTTATTGGGCCTTTTAATTAAAAAAATTAATTA
AAAAAATTACTATACATTAAAAAAACAATTTATTAGTAAAATAATTGTTTATTAATGGACAATAAAATATTTATTTTAATGTCTAAGAGACTTTATG
TGATTTACTTTTTTTTATTTGATTAAATCTATTATTATTATTATTCCTTTACTTATTGCTGTTGCATTTTTAACTTTAGCAGAACGAAAAATTTTAGGGTA
TATGCAAATAAGAAAAGGACCAAATGTTGTTGGAGGGGGGCTTCTTCAACCTTTTGCAGATGGGGTGAAATTATTTATTAAAGAAATGATTCTTCCC
CATCAAGCAAATAAATTTGTTTATCTTTTGGCTCCAGTTATATCTTTTACATTAGCTTTCTTTGTTTGAGGTTTTCTTCCTTATGAGAAAGGAGTGAGTA
TTAGTGATTTTAAAATTAGTCTTTTATGAATTTTGGCTATTTCTTCTATTAGTGTTTATGCAATTTTAATGTCTGGATGAGGAAGCAAGTCTAAATATG
CTTTTTTGGGTGCTATTCGGGCGGCGGCTCAAATGATTAGTTATGAGGTTTCAATTGGGCTAATTCTTATTTCGGTTTTATTATGTGTTGGTTCTTTAAG
TGTTACTGAAATTGTTTTAGCGCAAAATAATGGTATTTGGTTTTTTTTTCCACTATTTCCTGTTACAATAATGTTTTTTGCTTCGATTTTAGCAGAGACT
AATCGTGCTCCTTTTGATTTAACAGAAGGAGAGTCAGAGCTTGTTTCGGGATATAATGTTGAGTATGCTTCGATGTCTTTTGCTTTATTTTTTCTTGCT
GAATATGCACATATTATTTTAATGAGTTGTTTAACAACGATTTTTTTTTTGGGAGGATGGCTTTCTCCGATTCCAGATTTTAAGGGTGGGGCTGGATG
GTTTGGTTTTAAAGTTGTTTTTATTATTTTTTTTTTTTATTTGAGTAAGGGCTTCATTTCCTCGAATTCGATATGATCAGCTTATGGCTTTGTTATGAAAAG
GGTATTTGCCTTTAAGTTTAGGAATGGTCATTTTTGTGGCTAGTGTTCTCTTTGGGTTTAATGGCCCCCCTCCTATTTAAAATATTTTAATTAATTATTA
TGAAAAAAAAAAAAGAACTAACAGATTTGCAAGTGCCATTACGAAAAAAAGATCCTATTTTATTTTTAATCAACGGATTTTTAGTTGATTTGGTTTC
TCCTTCTAACATTACTTATTTATGAAATTTTGGATCTTTATTGGGATTGTGTTTGATTTTACAGATAGTCACTGGGTGTTTTTTGTCTATGCATTATTGTT
CTGATGTTAATTTTGCTTTTGCTTCAATTGGTCATATCATGCGAGATGTTAATTATGGGTTTTTATTAAGATATCTTCATGCTAACGGTGCTTCTTTGTT
TTTTTTTTGTCTTTATGTTCATATTGGTCGAAGTTTATATTATGGGGGGTATTTAAAATTTCATGTTTGAAGCATTGGAGTTGTTATTTTTTTATTAACAA
TGGCGATTGCCTTTATGGGCTATGTTTTGCCTTGGGGACAAATGTCTTTTTGAGGGGCAACTGTAATAACTAATTTATTGTCTGCTATTCCTTATTTTG
GGATTGATATTGTTCAGTGAGTGTGAGGCGGGTTTAGTGTTTCAAATGCAACATTAAATCGGTTTTTTAGTTTACATTTTTTACTTCCTTTTATTTTAGT
TTTTCTTGTTATTCTTCATTTAGTTTATTTACATGTTGATGGGTCTAATAATTCAACAGGATTAAATTCTTCAATTGACGGAGTTTCATTCCATACTTTTT
ATACATCAAAAGATTTTTTTGGTTTTTTTTTTCTTTTTTTTTTATTTTGTTTTTTTGTTTTTTTTTTGCCAAATTTATTAGGAGATGCTGAAAATTTTATTCA
AGCGAATTCTTTGGTTACTCCGGTTCACATTCAACCAGAATGATATTTTTTATTTGCTTATGCAATCTTACGTTCTATACCAAATAAATTAGGTGGGGT
TATTGCAATGTTTTGTAGTATTCTTATTTTATTTTTTTTATCTATTTTACATCAAAGTCTTTTAAAGGGGCTTTTTTTTCGTCCTTTAGGTCGAATTGCATT
TTGGTTTTTAATTATTGATTTTGCTTTATTAACTTGAATTGGATCACAAGTTGTAGAAGAACCTTTTATTTTAATTGGTCAAATACTCTCTTTTTTTTATT
TTTTTTATTTTTTAGTTTTAATACCAGTTTTGGGTATTATTGAAAATCAATTATTAAATAAAAAATTAATGAAATTTTTGTTTTAGGGGGTTTTCTTTTGA
GCTTCGTGGTTTTAAGTCTTCGTTATTTTCTTTTAAAACTTTCTCTTTTTTATTTATTATTATTTTTTTGTTTTTTTTTTTAAAATTAGAATGGGCAAAGCT
TTTGGTTTTAATTGGTTCTTTTGCTATCTTTCTTTTATTTGGACCAAAAAAAGAAGAACAAACAGATGTGCCTATTTTAAGTTTAATTATTGTTTTTGGA
GTTTTTTGTTTAATTTCTTCAAGTAATTGACTTTCTATTTATTTAACCATTGAACTTTTTACTCTTTGTTTTTTTATTTTGATTGCTCGAGGGTCTGGTTAT
AGTGTGGAAGCAGGATTAAAATATTTTATTTTGGGTGCTCTTTCTTCTGGTTTATTTTTATTTGGGTGTGCTTTATTATGTGGTATTGGGGCTAATATAC
ACTTTTCTCAKATAGAACTTCTTTTTAATTCAAAACAAGTTTTTTCTGCTGTTTCGATACCAATTGGGTATCTTTTAATTATTGTTGCTCTTTTTTTTAAA
TTATCAGTTGCTCCTTTTCATATGTGAGTTCCTGATGTTTATGAGGGGGCACCTACTAAAATTGTTCTATTATTGGCTATTGTTCCAAAAATAGGGTTT
TTTTCTCTTATAATTTCAATTGGATTGCCTGTAAATTTTTTTTTTTTTAGGGATTCTTTTTTCTTTGTTTGTTGGAGCCTTGGGTGCTTTAAATCAAACTAA
AATAAAACGACTTTTGGCTTATAGTGGGGTTGGTCATATGGGCTTTATTTTATGAGGTTTGGAAAATGGTTCTTTTGAAAGTTTACAAGCTAGTCTTG
TTTATCTTTTTATATATATTGTGATGACAATTTGTGCTTTTTCTATTATATTAAGTTTTAATGTTTATAAAAAATTGCTTGTTGAATTTAGTGGACTTTCT
CGATACTTACCTTTTTTTTCGATTACTTTAGGTGTTCTTTTTTTTTCTATTGCAGGAGTTCCCCCTTTTGCTGGATTTTTTGGAAAATGATTTATTTTGTTA
TCTGGAATTCTTTCTAAATCATATTTTATTTTTTTTTTTGCTGTTTTTTGTTCTGTTATAGCTGGGGTTTATTATATTCGAATTATAAAAATACTTTTTTTT
CAAAAAAATTCTTTTCTTTTAATTACAATTAAAGCTTTAAAAAAAGAATCTAAATTAAATTTTAAAAAAGTTTTTTTAATTGGTTTTTGTTTTTATTTTA
TTCTTTTTTTTTTTCTTTCTCCACATTTTCTTTTTTTTTTTTTTTCTCAAATAATTTTTGATTTATTTTAAAATGGAATTTTTTTCTTTTTTTTTTTATTTTAGGG
ATAATTGGCTCAGGAGTAATGGTTGTTTCAGCGTTAAATCCTGTTCTTTCTATTTTTTGATTGGTTCTTGTTTTTATAAATTCTGCAGTTTTTTTCCTTTT
ACTAGGAATAGATTTTCTTGCTTTGATGTTTTTACTTATTTATGTTGGGGCAATAGCTATTTTATTTTTATTTGTTATTATGTTATTAAATCTAACTGACT
ATCCTCCGGTTTTAAAAAGAGAGGTTGATATGACAAATTATATACCAATTGGATTTATAATTGGGATTTTTTTTTTTTCAGAAATTGCTTCAAGTGGA
TTATTTTTGGGTTCTTTTCAAATAGAGAATTGAGACCTTTCTTTTCCTTGGTTTCTTATTTCTTATCATAATATCGAGGCCTTAGGGCAGATTTTATATG
TTTCTTGTTTTTGTTTATTTCTTTTGGCCAGTTTTATTTTATTAGTTGCTATGATTGGTGTGATTGTATTAACTCAAGAAACAGAATTTTTAAGTAAAAA
ACAAGATCTTTTTTTTCAAATAAACAGGTAATGAATAGTTCTTCTTATTTTGAACAATTTAATATAGTGTGATTGTTTGGTTTTACGAATTCAACAATA
ATGATGACTTTTGTGATTATTGTCGTTTTATTACTTTTTAAAGGAATTGAATTAATTCCAAAAAGATGGCAGTCGGTTTATGAATATCTAGAAAATTA
TTTTTATTATATAACAGTGCAAAATTTAGGTAATGTGGGTTTAATATATTTTTCTTTTATTGTTTCACTTTTTGTTTTTTTAGTTTTTTTAAATATTTTAGG
TTTATGCCCCTACGTCTTTACTCCGACAACTCATATAATTGTTACTCTTGGATTTTCTTTTTCTATTGTTATTGGAGTAACTTTTTCTGGGCTTTATAAAT
TTAAAAAAGATTTTTTTAGTATTTTAATGCCGAGTGGGGCTCCTTTGATTTTGGCACCACTTTTAATTTTAATAGAAACAGTAAGTTATATCTCTCGGG
CTGTTTCTTTGGGGATTCGTTTAGCCGCGAATCTTTCTGCCGGGCATCTTTTATTTGCAATTTTGGCAGGATTTGGTTTGGTTTTTAAATTTGCAATGTC
TATAATGGTGTTTATTACGCTATTAGAGGTGGCAGTTGCTATAATTCAAGCCTATGTGTTTTGTCTGCTCACATTAATTTATTTAGCAGACACAATTTT
TTTACATTAAACATGACCGGATTAGAAATTTTTTTATTTACCATTGTGGGTGTTTCAATAGGGTGTCATTATGGGTTTGTAGTGTGTTGATATGGTCAT
TCCTCTCAGAGCTGACTTCGGCATCAAATGGATCTTCCAGAAAGCCTTGAATACGTCCATCCTTGAAATTATTTTGGTTATGTGCGTAATCATTATAT
TGCTCGGAGTATTGGGGTACAAGTGGATGTAGAACCAAATGAGGCCGGAGGTGTCTCTGAGAGATCTGGAGAAAACAATGGGGCCTTAAGTGGGA
TTAGAGTAGAAAGTTTAAGTAGTTGGAGTGAAAATAGAATTCGGGGATTAAGTGATTCACATAGAACTAGTGGATTAAGTGATTTAAGTACAATTG
GGGGATCAAGTGATTCAAGTACAATTTGGGGATTAAGTGATTCAAGTAGAATGCGGGCATTAAGTGATTCAATTAGGATATCAAGTGATTCAAGCA
CAATTCGAGCATTAAGTGATTTAAGTATAATTAGGGGATCAAGTGATTCAATTGGGGGATCAAGTGGTTCAATTGGGGGATCAAGTGATTCAAGTA
CAATTAGGGGATCAAGTGATTCAATTAGGGGAGGAAGTGATTCAATTAGAGGATCGAGTGATTCAAGTACAATTAGAGGATCGAGTGATTCAAGT
ACAATTAGGGGATCGAGTGATTCAAGTACAATTAGGGGATCAAGTGATTCAAGTACAATTAGGGGATCAAGTGATTCAGGAAGCGAAGACGAAAC
GTTAAGGGCAGTAAAGGTAGAAACACATTTAGGTGGTGACCAGTTAGCGTGTACAAATCAAGGTGGGGTTTTAAGAGAAAACAAGGTAGAAATTG
ATGAGGCAGTTGACTCGACTCTTAGTTCGATCCCTTTTGAGGTTGAAGGGATGATGGTTCATGTGTGAAATCAATTTGACATGGTGTTTAACATATTG

GCAGATAAGCCCCTGGTAGGCTGCATTTTTTTATCTTACCGGCTTTTAAAAATAACAAATCAAATGCCAAACCCACGGGTTTATAGGTGATTTATTTT
GGTATATATTTTATTAATCTTTGTTCTTTTAATGGTTTAGTTAACGTATGGTCTCTGGCAAGCAGATTTGGGGGGTTTAGAAAGGGAGCTAAAAAGTT
TTAAAATGTTTCTTTGTATTTGAATTAGCTAAAAATCAATAAAATTTTTATTTAAAGAATTTCTTTTGTGTTTGTTATTGTAATGGTTCTTATTTATTTAA
TTGTTATTATGGCATTAGTTAACATCATAGGGACAGCGAGGGAGAGAAGATGTGTTTTAAAAAAGCGGGGTTTAGAGTGGTCTTTGGCTTTATTGTT
TAGTACTTTAGTTTTTTGGGGTGGATTTGACGGAGAAAGTCATTTTCAATTTTTTAGTTTAGTAGAATGAAATATATTTTCTACTTTAGATTGAGGCCC
GATTGTTTTTGCAGTCGATGGTGTTTCTTTGGTTTTTTTACTTTTAACGACTTTTTAATTCCAATTTGTATTTTAATTAGCCAAAAATCAATAAAATTTT
TATTTAAAGAATTTCTTTTGTGCTTGTTTTTTTTAGAAGTTTTATTAATAGGTGTTTTTATAGTGTTTGATCTTCTTTTGTTTTATCTTTTTTTTGAGGGGA
TATTAATACCAATGTTTTTTTTAATTGGTATTTGAGGCTCTCGAGAAGAAAAGGTTCGTGCTTCTTTTTATTTTTTTTTTTTTTACTTTTATAGGCTCTCTC
TTTTTTTTTTTTATAATACTTTTTTTATATCAAAGGATTGGAACAACAGACTATTTTCTTTTACTTAATATTAAATTATTTTTAAATATTCAAAAATGAG
CCTTAGTTGGGATTTTTCTTAGTTTTGCGGTGAAACTACCTCTTATTCCATTTCATATTTGATTGCCACAAGCACATGTTGAGGCTCCTGTTGCGGGCT
CTGTTATTTTGGCTGGAATTTTATTAAAATTAGGGGGTTATGGCCTTTTGCGTTTTTCTTGACCTCTTTTTCCGGGGGCTTCTTTATATTGGTCTCCAGTT
ATTGTTTTTTTTAGTGTTGTTGCTGTTGTTTATGGAGGCTTAATGACATGTCGTCAAATTGATTTTAAACGGCTTGTTGCTTACTCTTCTGTTGCTCATAT
GGGACTTGTGCCTTTGGGTCTTTTTACACATGTTATAGAGGGGTTAATTGGGGCTCTTTTTTTAATGTTGGCGCACGGATTTGTTAGCTCTGCTCTTTTT
ATTGGAGTAACTTTTTTGTATGATCGCCACCATACTCGTTTAATAAAAATATTATCGGGGTTTGACTTTGACAATGCCTCTTTTTGTTATTACAATGTTA
ATTTGTCTTTGGCGAACATGGGTTTTCCTCTTAGTTGTAATTTTGTTGGAGAATTTTTTTCTTATTAGCAGTCTTTCAATATCATTATGGAGTTGGAA
TGTTTGTTATTTTAGGGGTTCTTTTTTCTGCAATTTATTCTCTTAGTCTTTTTAATCGTATTTCTTTTGGTGGAGGATCTAATTATTTACTTTTTAATAGAG
ACTTAAGTCGACGAGAGATTTTTGTAATTTTTCCTTTTCTTTTAATTATCTTAGGGGGGGGGGATTGTGCCTTTTCCTATTATTGATTTAATTAAAAATAG
TCTTGTTTTTAGCCCAGGGTGTTAGTCCTGGGGTTTTAGGGAAAAAAAAAATATTTAGTCTAGGTTATACATGCTAGTGTATGCGAACAGGTGAGGAT
AAAATAAAAAAATTTTTTTATTTGTTGTATTAAAAAAAGAAAAGTTTTTTATTCTTTTTTTGAAGATGCATAGTTTAACCACATTTTCACTGAAACA
AAAGAAAACCTTTGGCAGCAGTAATGGATTTTATGTAATATACGAAAGTAAGACATAGGTGGGATAAAAAAAAATCTGTCAAATATAAGTGCCAGC
AGACGCGGTAAAACTTAAGGATTTATTTTTTAATAAAAAGCAAAAAGCGTGTTAAGGATTTTTAAAAAAAAAAAAAATAAATAGAATTTTTTTCGTA
ATTGTGAAATGTTGGAATGAAAAAAAGAATTTTTTATATGAAAATAATTTATTTTTTTTTTCTTAAATACGAAGGTTTTGGGAGCAAACAGGATTAG
AGACCCTGGTAGTCTATACAGTAAAAGAATATCGCTTGAGTAGTACGGTCGCAAGATTGAAATTCAAAAGACTTGGCTGTTCGGTTGTTAATTAGA
GGAGCGCGTCGCTTAATTCGATAATCCGCGAGAGACCTTACCGAAGTTTGAATTTATTAACAGGTGTTGCATGGCCGTCGTCAATTTCTGTTTGAAA
CAAAAAGGGGTTTAATCCTATCAAGGTTGAAGTCAGGTATTATGACCCTTATATTTCGGGGTTAGGCGCGCTACATTTTTCTCTTAGAAAAGGAGGG
GTTCGGATTGTTTTTAAAATAAGATAATGAAGTTGGATTTAATAGTAATTGAAAAGTAGTGCGTTTCAATGAACGTGAAAACAATGTTAGTACAAAT
AGCCCGTCGCCAGCCCAGAAATAGGCTGTAAGTCGTAACATAGTAAGAGTGAGGGAACTGGCTCTTGATGCAGTTTCATTCGTATCATTTAGTTGA
GCCTTCTCCATGGCCTTTTGTTGGAGCAATTGGCTCTTTTTTTATTACTGTTGGTGCAGTGGTTTTTTTTCATTATGGGTTTAGTTTTTTTTTATATTTGGG
GCTTTTGGTTGTTGTTGGAGTTATGTTTGTTTGATGACAAGATGTTATACGAGAATCAACTTTTCAAGGTCACCATTCTTTAATTGTAAAACAAGGGA
TTAAATATGGTATGATTTTATTTATTCTTTCAGAAATTTTGTTTTTTTTTTCTTTTTTTTGGGCTTTTTTTCATAGTAGTTTAGCACCGGTTGTTGAACTTG
GAGTTGTTTGGCCTCCACAAGGAATTGTTGCATTAAATCCCTTTTCTGTTCCTTTATTAAACACTGCTGTATTATTAAGTTCTGGGGCAACGGTTACAT
GGGCGCACCATGCAATACTCTGTGGTTTAAAAAAAGAGGCTCAACTTGCTTTATTTTTAACTCTTTTTTTGGGTGTTATGTTTACGGGGTTGCAAGCA
TTTGAATATTATGAGGCGCCTTTTACTTTATCTGACTCTGTTTATGGGGCCACTTTTTTTGTTGCGACAGGGTTTCATGGACTACATGTTATAATTGGTA
CTACTTTTCTTTTTATTTGTTTTTTACGTTTACTCTCTAATCAATTTACTCGTCGTCAACATGTTGGGTTTGAGGCCGCGAGTTGATACTGGCACTTTGTC
GATGTGGTTTGATTATTTTTATATTTATGTATTTATTGATGAGGCTCATAAAAACTTCTTTAATGCAAAATCTTTTTGTTTTTACTTTTTTTAGATATTTT
ATAAATGTTTTGGCTTGGTTGTCTGTTCTCTGGTTTAATCATCTTTATTATTCGGACAGTCCAGAGACTTGACTTTTAGAGTTTCAAGATGTTGGGGAT
CCTATTGTAGAAGAAATTGTTTTTTTTCATGACCAAGTTATGTTTTTATTAATTATTATAGTTACTGTTGTTTTATGACTTTTTGTTGAATCTTTTAAAAA
TAAGTTTTATGATCGTCATTTAATCGATGGTACATTTTTAGAAATTGTTTGAACGATTATTCCTGCTGTTATATTGATTTTTATTGCATTGCCCTCTCTT
AAATTATTATATTTAATGGATGAGGTTATTTCTCCAGCTTTGACAATTAAAGTTATTGGGCATCAATGATATTGATCCTATGAATATTCTGATTATGA
AGGGGATACGTTAGGGTTTGATTCTTATATGGTTCCTACTTCGGATTTAGTTTCTGGAGAGAATCGTTTGTTAGAAGTTGATTATAAACTTTTAATTCC
TATTCAAACACATATAAGATTTTTAGTTACTGGGGCAGATGTTTTACATTCTTTTGCAGTGCCTTCTTTGGGGTTAAAAATTGATGCGGTTCCGGGTCG
TCTAAATCAAACTGGTGTTTTTATAAAACGAGCCGGGGTTTTTTTTGGACAATGTTCTGAAATTTGTGGGGCAAATCATTCTTTTATGCCAATTGTGAT
AAAGGGAGTTGGTTTAAATGAATACGTTCAATATTTAAATTATTTAAAATGTATTATAAATACTTAGTTATTATTATTATTTTATTTTTATTGGGGAGT
TGGGGTATAATTTTAAATAGAGGACATTTTATTATTATGCTTGTTTCTATTGAATTAGTTTTATTATCAACTTTTTTTTTTTTTTTAATAAGCTCTAAAGA
AATAGATCTTTTAATAGAACAAATTTTTATGATTATGGGATTAACTATTGCTGCGGCAGAATCTTCAATTGGTTTAGCTATTTTAGTTGCATATTATAG
AATTCGAGGAACAATTGTTTTAAAATCTTTTAGTTCTTTGCGAGGATAATGATTAATCTTTTTATTTTTTTTTTTCTTATTTTTATTTTGGCGGGGTTATT
TATATTTTTTTCTTTTTTGATAGGGGAAAAAAATTCCAGATCGAGAAAAGGTTTCTGCTTATGAATGTGGTTTTGCTCCCTTTAATTTTTTAGGGCACCC
TTTTTCAATACGTTTTTTTTTAATTGGTATTTTATTCTTGATTTTTGATTTAGAAATCTCTTTTTTTTTTCCCTGATGTGTTTTATATAATTCGACTGCTCCG
TTTGGGTTTTGAACTATGATAGGTTTTTTTTTTGTATTAGTTTTGGGTTTGATATACGAATGAGTAATGGGGGGGTTAGAATGGGAGTAAAAAGAGAG
TAAAAGAAAAAGTCCTATCTATTATGAAAATAATAGAAATTTTATACCAACTCCGGTTTCTGCTTTAATTCATGCTGCAACGATGGTTACTGCAGGT
GTTTTTTTATTAATTCGTGCTTCTCCTTTATTTGATGTTGTTCCTCTTATATTAATTATTATTTCAATCGTTGGGGTTTTAACCGTGTTTATTGCAGGGAC
AATTGGTTTAGTTCAAAATGATTTAAAAAAAAATAATCGCTTATTCTACTTGTAGTCAATTAGGTTATATGGTTGTTGCTTGTGGGCTTTCTCATTATGC
AATTAGTCTTTTTCATCTTATGAATCATGCTTTTTTCAAAGCTTTATTATTTTTGAGTGCAGGGTCTCTTATTCATGCGGTAATTGATGAACAAGACAT
AAGAAAAATGGGGGGTTTATTATCTTTTCTTCCTTTGACTTATGTTTTTTTTTTGATAGGCTCTTTTTCTTTAATGGGGTTTCCTTTTTTAACAGGATTTT
ATTCAAAAGATTTAATTTTAGAATTTGCTTTTGGACAATTTTATTTAATTTTTGTGTATTGATTAGGTTGTTTTTCTGTTTTATTAACAATTATATACTCT
ATTCGTTTAATTTATTTAGTTTTTTTATCCAATATTAATTTAAAACGAGCAAACATTTTTTTTTCTTAAAGAAGGAGAATTTTTATTTTTAATTCCTTTGG
GTATATTAACTTTAGGAAGTGTTTTTTGAGGCTATTTAAGTAAAGAAATAATTTGGTCTTTTCAAATAGATGTTTTTTCAATACTTTCTTTAAAAATAA
AAATATTTCCAATTTTATTTTGTTTTATTGGACTTTTGGAACGATATTTTTTTTTTTTTTTTTTTCTTCTCAAATTTTTGGCTATCCCCTTCAGGTGATCA
GATCTTCTGTTTTTTCTCTTTATAATTTTTTTGGTTCTGCTTGACAAATAAATTTTTTTTTTAATTTTTTCTTTATAAAAAAAATATATAAAATAGGACAT
CTAATTACTAATTTAACAATTGATAAAGGTTTATTAGAGGTTGTTGGGCCTAGGGGTGTTGTTCAATTTTTTATTTTTCAAACTCAAAAGTTGAGTAGT
TTACAATCGGGGTTAGTATTTAATTATGCTTTAGTTTTTTTTCTTGGGATACTTTTTTTAATTTTTGCACTTTAATTTAAATTTTTGAAGGGGTTAAGTTA
AAGTAAACTGTTGATCTTCAAAATCAGCTATGTAGGTGCAAGTCCTACACTCTTTGAGTGCCTCAGTTGAAAGTCGGCTTTTATAAGGTACAATATT
GATGGTGTTTTTCTGTTTTGTTTTTATTGTTAATTTTTTTTGAGGTTGTTATTTTTCCTTTAATAAAAAAATAATTGATGTATAAGAAAGTTCTTAATGAAG
TGCAATGGCGCCATCTTAACAAAAAGTTGGTTACAAAAAGAACTTTATAAAAAAAAAAAAAAAAAGTATGGTGTAATACTAAACTTTTTTAAATGATG
AGTTATATTAAATATTTATGAAAAAAATGGGCTAGTATTGGGTTTATTAGTAAGTTGCAAGTTTTTTTGATGAGGTATAGATTGAAAATTATTTTGAT
TATCGGTGGTGTAATCCTTCTCGTTATAGGAATACTCCTAGCTGTCCTAGGAGTGGATTTCGAGGGGGAGTTTTTATAAATGTTTTAAAAAAAAAATTGT

TTGTTGACGCTGCGGGTCCGCCCCGTAAACGTGCTAGGGTTGAAGAAGAACGATCGTCAAATATGCCCGGGAATATTGAAAATGAAATCGTGGGC
GGAGCCGAAAATGAAGCCGCAGGCGGAGCCGAAGATGGGGCAGCGGGCGGAGCCGAAAACGAAGAGCTACCGGCGGCGTTTGCACCTTTAGCC
AACCCAGTGAGTACCGGAGTTCGAGAAACCGAGGGAGATTTGGGTTATTCAGATGAGGTTTGGGATGCATTAAATGGATTTCAGAAGATCAAGAA
GACGAATTAGTAGAGATGCTAATTGGGTGAGCAAACAGTGTAACGGCCGGAGAGTCAAACGAGGATATAGTAGATGTGCTAGGGGAGTGGGCAG
AGGGTAACATCCCCGAAGATTCAGGCGACGAATCAGAGGAGAACGCGTCTGAAGATTCAAACGACGAATAGGCGGAGGCCCCCATTTATTCAATA
TGTAAGAGATCGGGGGGGGGTTGTGGGACCTCGCCCCGGGTATTTGAACGATATGGTCTCTAATTATTGAATTCGATGGGGGTTTTCAACCAACCAT
AAAGATATTGGTAGTTTGTATTTAATTTTTGGTGGAGGTGCTGGTTTAATTGGAACGGCGTTTAGTATGCTTATACGACTAGAGCTTTCTGCGCCCGG
AGCAATGTTAGGAGATGATCATCTTTATAATGTAATTGTTACAGCGCATGCTTTTATTATGATTTTTTTTTTGGTTATGCCGGTTATGATTGGGGGGTT
TGGTAATTGATTGGTCCCATTATATATTGGGGCGCCGGATATGGCGTTTCCCCGACTAAACAATATTAGTTTTTGACTTTTGCCCCCTGCGCTTTTTTT
ATTATTAGGCTCTGCTTTTGTTGAACAAGGGGCGGGGACGGGGTGAACAGTTTATCCTCCTCTTGCTAGTATTCAAGCACACTCCGGATGTTCGGTTG
ATATGGTTATTTTTAGTCTTCATTTAGCTGGGGTTTCTTCTATTTTAGGTGCTATAAACTTTATTACTACAATTTTTAATATGCGAGCCCCGGGTGTGTC
TTTTAATAAACTACCTTTATTTGTTTGATCTATTTTAATAACAGCTTTTTTATTACTTTTATCTTTACCTGTTTTAGCTGGTGCTATTACTATGTTGTTAAC
AGATAGAAACTTTAATACGACTTTTTTCGATCCAGCGGGTGGCGGGGACCCAATATTATTTCAGCATCTATTTTGATTCTTTGGGCATCCAGAAGTTT
ATATTTTAATTTTGCCTGGTTTTGGTATGATTTCTCAAATAATCCCGACTTTTGTTGCTAAAAAACAAGTTTTCGGGTACTTAGGAATGGTTTATGCCA
TGCTTTCTATTGGGCTTCTGGGATTTATTGTTTGAGCTCATCATATGTTTACTGTTGGGATGGATGTAGATACAAGAGCATATTTTACTGCTGCTACTA
TGATTATTGCTGTGCCAACTGGGATTAAAGTTTTTAGTTGGTTGGCAACTATTTATGGAGGTGTTCTTAGGTTAGAGACTCCAATGCTTTGAGCTATG
GGGTTTGTTTTTTTATTTACAGTTGGTGGTTTAACTGGGGTTGTATTAGCAAATAGTTCTCTTGATATTGTTCTACATGATACATATTATGTAGTTGCGC
ATTTTCATTATGTTCTTTCTATGGGGGCTGTTTTTGCTATTTTTGGGGGATTTTACTATTGAATTGGAAAAATAAGTGGTTATTGTTATAATGAATTTTT
TGGGAAAGTTCATTTTTGATTAATGTTTATCGGGGTTAATTTAACTTTTTTCCCTCAACATTTTTTAGGTTTAGCAGGATTTCCAAGACGATACTCGGA
TTATGCAGATGCTTTTTTGGGTTGAAATTTAATAAGTTCTTTAGGGTCTATTATTTCTATTTTGAGTGTTGTTTGGTTTTTATATATTGTTTTTGATCTTTT
TGTTACAGAAGAAAAATTTTTGGGTTGAAAAGAAGGATTTTCTTTAGAATGAATTCATTCTTCTCCCCCCTTATTTCATACTTATGAGGAGTTGCCTTT
TGTACAAAAATAAATAGTTTTTAGAATAGTGCCGGGTTTATATACCGGTTT

Supplementary Data S2. Translated mtORF sequences of Pocilloporidae corals

>EU400212.1 (NCBI Reference Sequence: NC_011160.1)_*Madracis mirabilis*

MPDLEIFFLCLLSGSVVINYSLCLCWYSARVQRWLLENMQVEPQEDGSYPIIHFWTFMNHVRRLNNTREVAQREALEVEGPVSEPEEVVIHIYENPLARGDVVLRNVYEGP
VSEDEGATSQEIRGGVMETSLYERKSELIEPPLSSPSFEGGGWMSHMPLEPIFGLIPFECVDILLHGPLISLIFLYLRLLCYADQMVNPRAFRLFLLGYFLFLVVILVLI

>EF526303.1_*Pocillopora_eydouxi*

MTAVEIFLCTIIGISLTVNYGFGIGWYSSSSQAWLRHQMGIDGTDMVHPWAYFNYVRQHSIARNVGVQVNLDSTEGPNDVGVQVNQESEQSVDVVFIELEGRRVPVRGV
NLHVERRGYLDNRSNQEGVENSLGDQFEDVAFLGGSTSTLTPESVNRGAPSGNIRNVLSDSLIERRGQDQTLSEIKVETPPGASRGGRWASINQGEALRENKVEIGVLREN
KVEIDEVVGSTFSSIPLEVEGMMAHVWNQFDMVFNILADKPLVGCIFFSYQLLRITNQMPNPRVYRWFALVYLLFIFVLLMV

>EU400213.1_*Pocillopora_damicornis*

MTAVEIFLCTIVGISLTVNYGFGIGWYGSSSQAWLRHQMGIDGAERVHPWAYFNYVRQHSIARNVGVQVNLDSTEGPNDVGVQVNQESEQSVDVVFIELEGRRVPVRGV
TLHVERRGYLDNRSNQEGVENSLGDQFEDVAFLRESTSTITPESVNRGAPSGNIRNVLSDSLIEKRGQDQTLSEIKVETPPGASRGGRWASINQGEALRENKVEIGVLRENK
VEIDEVVGSTFSSIPLEVEGMMAHVWNQFDMVFNILADKPLVGCIFFSYQLLRITNQMPNPRVYRWFALVYLLFIFVLLMV

>EF633601.2_*Seriatopora_caliendrum*

MGELEIIFYIIFMVSSGLNWGFVVMWYNSSSQRWLRHQMGTDGWVHPHNYISYVRRHGIAERQVGREGPTEAMYVESGGRPRNWGEEDSESESIANLRECVDRAIRQLES
RENLREGETSALSESESRDNLRESVANALSESESRGDLRESVTRVLRESRSRDETLRGGEAETYVCDGRVNIDEAVESTLSSISFEVGETMVHVWNQFDMLFNILACRPLVGC
IFLSYQLLKIANQMPNPRVYRWFVLVYILLIFVFFLP

>EF633600.2_*Seriatopora_hystrix*

MSELEIIFYIIFFVSAGFNWGFFVMWYNSSSQRWLRHQMGTDDWVHPHNYISYVRRHGIAERQVGREGSTEVMYVESGRRPRNGGLEDGEPESIENLRECVVRALRQLESR
ENLREGETSALSESESRENLREGETSALSEAESRDNLRESVASALSESESRGDLRESVTRVLRESRSQDETYVCDGRVNIDEAVESTLSSISFEVGETMVHVWNQFDMLFNILA
CRPLVGCIFLSYQLLKIANQMPNPRVYRWFVLVYILLIFVFFFP

>*RS_LinB*

MTGLEIFLFTIVGVSIGCHYGFVVCWYGHSSQSWLRHQMDLPESLEYVHPWNYFGYVRNHYIARSIGVQVDVEPNEAGGVSERSGENNGALSGIRVESLSSWSENRIRGLS
DSHRTSGLSDLSTIGGSSDSSTIWGLSDSSRMRALSDSIRISSDSSTIRALSDLSIIRGSSDSIGGSSGSIGGSSDSSTIRGSSDSIRGGSDSIRGSSDSSTIRGSSDSSTIRGSSDSSTIRGS
SDSSTIRGSSDSGSEDETLRAVKVETHLGGDQLACTNQGGVLRENKVEIDEAVDSTLSSIPFEVEGMMVHVWNQFDMVFNILADKPLVGCIFLSYRLLKITNQMPNPRVYR
WFILVYILLIFVLLMV

>*RS_LinA*

MSGVEIFLCSLMGVSVCCNYAFMMAWYSQSSQRWLQHQMDLPENTEYVHPWTYYSYVRRHGIVHNGGVQVDQESNGADRPRDVIFIDLERRQVPLRGRRVFRVAEND
ESLCDNGNRLCHDEGLCDNDDRLCVHGESLCDDDSLCDNDEFQDGVLHLSKSASESESRGSLRESVIKALSESESRGSLRESIIKGLSDSRSEDEALRAVEVETHLSGGQLA
RTNQGEAVESTLSSIPFEVEGMMVPVWNQFGMLFNILGDKPLVSCIFLCYQLLKITNQMPNPWVYRWFILVYILLIFVFLMI

>EU400214.1_*Stylophora_pistillata*

MSGVEIFLCSLMGVSVCFNYAFMMAWYSQSSQRWLQHQMDLPENTEYVHPWTYYSYVRRHGIVHNGGGDRPRDVIFIDLEGRQVPLRSRRFFRVAENDESLCDNGNRL
CHDEGLCDNDDRLCVHGESLCDDDSLCDNDEFQDGVLHLSKSASESESRGSLRESVIKGLSESESRGSLRESVIKGLSESESRGSLRESVIKGLSDSRSEDEALRAVEVETHLS
GGQLARTNQGEAVESTLSSIPFEVEGMMVPVWNQFDMLSNILGDKPLVSCIFLSYQLLKITNQMPNPWVYRWFILVYISLIFVFLMV
