# Supplementary material for: Computational Characterization of the mtORF of Pocilloporid Corals: Insights into Protein Structure and Function in Stylophora Lineages from Contrasting Environments
Source: Genes (Basel). 2019 Apr 27;10(5):324. doi: 10.3390/genes10050324 (PMC6562464; doi:10.3390/genes10050324)

Supplementary Figure S1. Transmembrane domains and pore-lining regions in pocilloporid corals.

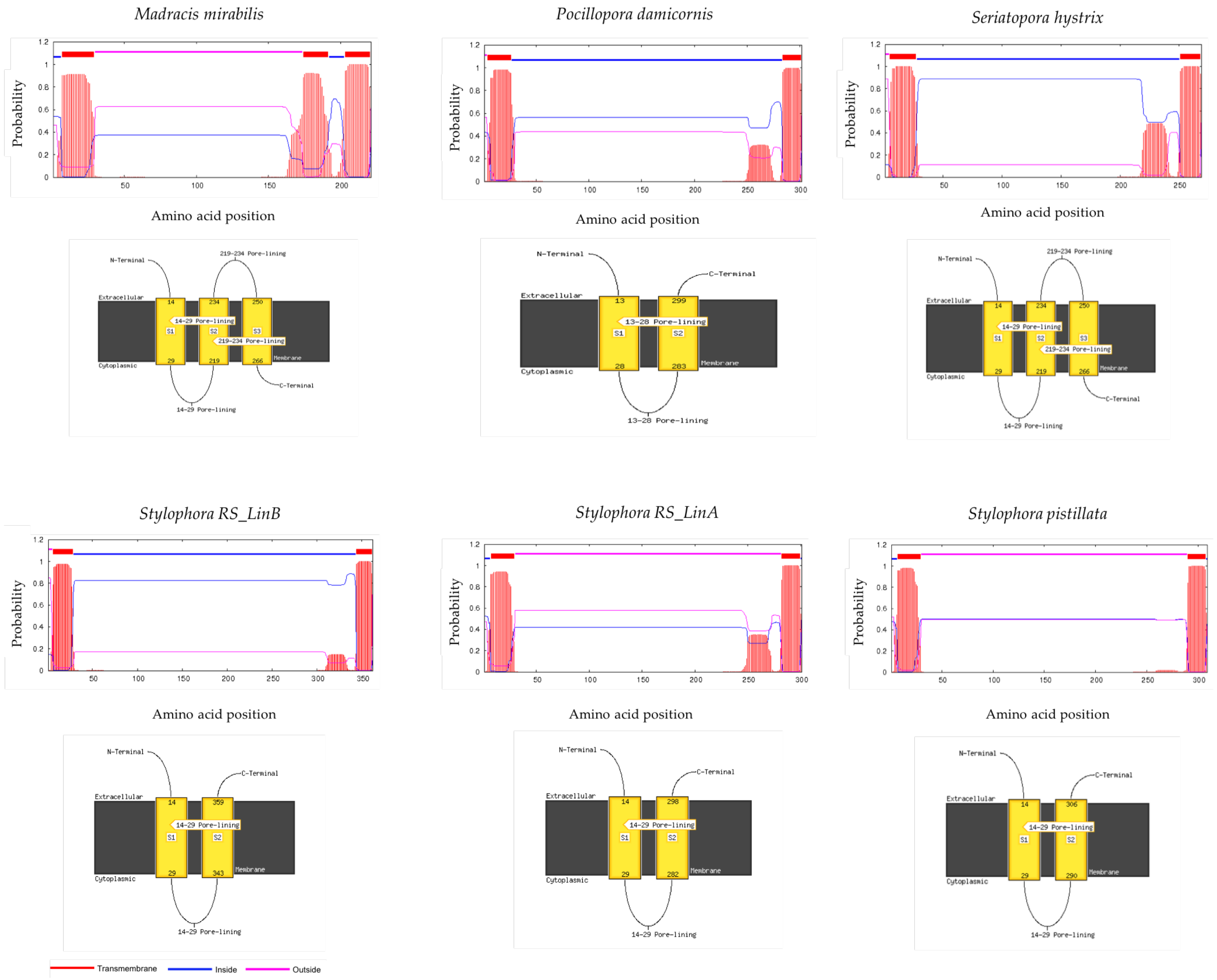

Supplement: Supplementary file 1 [file genes-10-00324-s001.zip › Supplementary_26042019/Supplementary_Figure_S1.pdf]
