# Supplementary material for: Computational Characterization of the mtORF of Pocilloporid Corals: Insights into Protein Structure and Function in Stylophora Lineages from Contrasting Environments
Source: Genes (Basel). 2019 Apr 27;10(5):324. doi: 10.3390/genes10050324 (PMC6562464; doi:10.3390/genes10050324)
Supplement: Supplementary file 1 [file genes-10-00324-s001.zip › Supplementary_26042019/Supplementary_Table_S1.pdf]

Supplementary Table S1. Accession numbers of mtORF sequences analyzed in this study.

Genera	Accession numbers	References*
Pocillopora	EF526302-EF526303	Flot and Tillier 2007 [1]
	EU374225-EU374261	Flot et al. 2008 [2]
	FJ424084-FJ424129	Souter 2010 [3]
	FR729326-FR729399	Flot et al. 2010 [4]
	HQ378758-HQ37861	Pinzon and LaJeunesse 2011 [5]
	JX624970-JX625077	Schmidt-Roach et al. 2013 [6]
	JX983175-JX994088	Pinzon et al. 2013 [7]
	KF583920-KF583950	Marti-Puig et al. 2014 [8]
	KF709240-KF709244	Schmidt-Roach et al. 2014 [9]
	KF985974-KF985980	Paz-García et al. 2015 [10]
	KJ690905-KJ690907	Paz-García et al. 2015 [10]
	KM215069-KM215131	Hsu et al. 2014 [11]
	KM610241-KM610280	Baums et al. 2014 [12]
	KT879922-KT880039	Gélin et al. 2017a [13]
	KX5389810-KX539013	Gélin et al. 2017b [14]
	KY587458-KY587472	Poquita-Du et al. 2017 [15]
	LC222444-LC222452	Nakajima et al. 2017 [16]
	NC009797-NC009798 (complete mitogenomes)	Flot and Tillier 2007 [1]
Seriatopora	NC010244-NC010245	Chen et al. 2008 [17]
	EU622126-EU622176	Flot et al. 2008 [2]
	HQ878445-HQ878586	Van Oppen et al. 2011 [18]
	KR150024-KR150046	Warner et al. 2015 [19]
	KX618663	Gélin et al. 2017b [14]
	KY987919-KY988112	Sinniger et al. 2017 [20]
	EF633600-EF633601 (complete mitogenomes)	Chen et al. 2008 [17]
Stylophora	FN674400- FN674440	Stefani et al. 2011 [21]
	JN558840- JN558909	Flot et al. 2011 [22]
	NC_011162 (complete mitogenome)	Chen et al. 2008 [17]
	EU400214 (complete mitogenome)	Chen et al. 2008 [17]

References

1. Flot, J-F.; Tillier, S. The mitochondrial genome of *Pocillopora* (Cnidaria: Scleractinia) contains two variable regions: the putative D-loop and a novel ORF of unknown function. *Gene* **2007**, *401*, 80–87.

2. Flot, J-F.; Magalon, H.; Cruaud, C.; Couloux, A.; Tillier, S. Patterns of genetic structure among Hawaiian corals of the genus *Pocillopora* yield clusters of individuals that are compatible with morphology. *C. R. Biol.* **2008**, *331*, 239–247.

3. Souter, P. Hidden genetic diversity in a key model species of coral. *Mar. Biol.* **2010**, *157*, 875–885.

4. Flot, J-F.; Couloux, A.; Tillier, S. Haplowebs as a graphical tool for delimiting species: a revival of Doyle's "field for recombination" approach and its application to the coral genus *Pocillopora* in Clipperton. BMC Evol. Biol. 2010, 10, 372.

5. Pinzon, J.H.; LaJeunesse, T.C. Species delimitation of common reef corals in the genus *Pocillopora* using nucleotide sequence phylogenies, population genetics and symbiosis ecology. *Mol. Ecol.* **2011**, *20*, 311–325.

6. Schmidt-Roach, S.; Lundgren, P.; Miller, K.J.; Gerlach, G.; Noreen, A.M.E.; Andreakis, N. Assessing hidden species diversity in the coral *Pocillopora damicornis* from Eastern Australia. *Coral Reefs* **2013**, *32*, 161–172.

7. Pinzón, J.H.; Sampayo, E.; Cox, E.; Chauka, L.J.; Chen, C.A.; Voolstra, C.R.; LaJeunesse, T.C. Blind to morphology: genetics identifies several widespread ecologically common species and few endemics among Indo-Pacific cauliflower corals (*Pocillopora*, Scleractinia). *J. Biogeogr.* **2013**, *40*, 1595–1608.

8. Marti-Puig, P.; Forsman, Z.H.; Haverkort-Yeh, R.D.; Knapp, I.S.S.; Maragos, J.E.; Toonen, R.J. Extreme phenotypic polymorphism in the coral genus *Pocillopora*; micro-morphology corresponds to mitochondrial groups, while colony morphology does not. *Bull. Mar. Sci.* **2014**, *90*, 211–231.

9. Schmidt-Roach, S.; Miller, K.J.; Lundgren, P.; Andreakis, N. With eyes wide open: a revision of species within and closely related to the *Pocillopora damicornis* species complex (Scleractinia; Pocilloporidae) using morphology and genetics. *Zool. J. Linn. Soc.* **2014**, *170*, 1–33.

10. Paz-García, D.A.; Hellberg, M.E.; García-de-León, F.J.; Balart, E.F. Switch between morphospecies of *Pocillopora* corals. *Am. Nat.* **2015**, *186*, 434–440.

11. Hsu, C.-M.; de Palmas, S.; Kuo, C.-Y.; Denis, V.; Chen, C.A. Identification of scleractinian coral recruits using fluorescent censusing and DNA barcoding techniques. *PLoS One* **2014**, *9*, e107366.

12. Baums, I.B.; Devlin-Durante, M.; Laing, B.A.A.; Feingold, J.; Smith, T.; Bruckner, A.; Monteiro, J. Marginal coral populations: the densest known aggregation of *Pocillopora* in the Galápagos Archipelago is of asexual origin. *Front. Mar. Sci.* **2014**, *1*.

13. Gélin, P.; Postaire, B.; Fauvelot, C.; Magalon, H. Reevaluating species number, distribution and endemism of the coral genus *Pocillopora* Lamarck, 1816 using species delimitation methods and microsatellites. *Mol. Phylogenet. Evol.* **2017**, *109*, 430–446.

14. Gélin, P.; Fauvelot, C.; Mehn, V.; Bureau, S.; Rouzé, H.; Magalon, H. Superclone expansion, long-distance clonal dispersal and local genetic structuring in the coral *Pocillopora damicornis* type β in Reunion Island, south western Indian Ocean. *PLoS One* **2017**, *12*, e0169692.

15. Poquita-Du, R.; Ng, C.S.L.; Loo, J. Bin; Afiq-Rosli, L.; Tay, Y.C.; Todd, P.; Chou, L.M.; Huang, D. New evidence shows that *Pocillopora* 'damicornis-like' corals in Singapore are actually *Pocillopora acuta* (Scleractinia: Pocilloporidae). *Biodivers. Data J.* **2017**, *5*, e11407.

16. Nakajima, Y.; Wepfer, P.H.; Suzuki, S.; Zayasu, Y.; Shinzato, C.; Satoh, N.; Mitarai, S. Microsatellite markers for multiple *Pocillopora* genetic lineages offer new insights about coral populations. *Sci. Rep.* **2017**.

17. Chen, C.A.; Chiou, C.-Y.Y.; Dai, C.-F.F.; Chen, C.A. Unique mitogenomic features in the scleractinian family pocilloporidae (Scleractinia: Astrocoeniina). *Mar. Biotechnol.* **2008**, *10*, 538–553.

18. Van Oppen, M.J.H.; Bongaerts, P.; Underwood, J.N.; Peplow, L.M.; Cooper, T.F. The role of deep reefs in shallow reef recovery: an assessment of vertical connectivity in a brooding coral from west and east Australia. Mol. Ecol. 2011, 20, 1647–1660.

19. Warner, P.A.; Van Oppen, M.J.H.; Willis, B.L. Unexpected cryptic species diversity in the widespread coral *Seriatopora hystrix* masks spatial-genetic patterns of connectivity. *Mol. Ecol.* **2015**, *24*, 2993–3008.

20. Sinniger, F.; Prasetia, R.; Yorifuji, M.; Bongaerts, P.; Harii, S. *Seriatopora* diversity preserved in upper mesophotic coral ecosystems in southern Japan. *Front. Mar. Sci.* **2017**, *4*, 1–9.

21. Stefani, F.; Benzoni, F.; Yang, S.Y.; Pichon, M.; Galli, P.; Chen, C.A. Comparison of morphological and genetic analyses reveals cryptic divergence and morphological plasticity in *Stylophora* (Cnidaria, Scleractinia). *Coral Reefs* **2011**, *30*, 1033–1049.

22. Flot, J-F.; Blanchot, J.; Charpy, L.; Cruaud, C.; Licuanan, W.Y.; Nakano, Y.; Payri, C.; Tillier, S. Incongruence between morphotypes and genetically delimited species in the coral genus *Stylophora*: phenotypic plasticity, morphological convergence, morphological stasis or interspecific hybridization? *BMC Ecol.* **2011**, *11*, 22.
